# Fauna Europaea: Neuropterida (Raphidioptera, Megaloptera, Neuroptera)

**DOI:** 10.3897/BDJ.3.e4830

**Published:** 2015-04-17

**Authors:** Ulrike Aspöck, Horst Aspöck, Agostino Letardi, Yde de Jong

**Affiliations:** ‡ Natural History Museum Vienna, 2nd Zoological Department, Burgring 7, 1010, Vienna, Austria; § Institute of Specific Prophylaxis and Tropical Medicine, Medical Parasitology, Medical University (MUW), Kinderspitalgasse 15, 1090, Vienna, Austria; | ENEA, Technical Unit for Sustainable Development and Agro-industrial innovation, Sustainable Management of Agricultural Ecosystems Laboratory, Rome, Italy; ¶ University of Amsterdam - Faculty of Science, Amsterdam, Netherlands; # University of Eastern Finland, Joensuu, Finland

**Keywords:** Biodiversity Informatics, Fauna Europaea, Raphidioptera, Megaloptera, Neuroptera, Europe, Taxonomy, Taxonomic indexing

## Abstract

*Fauna Europaea* provides a public web-service with an index of scientific names of all living European land and freshwater animals, their geographical distribution at country level (up to the Urals, excluding the Caucasus region), and some additional information. The *Fauna Europaea* project covers about 230,000 taxonomic names, including 130,000 accepted species and 14,000 accepted subspecies, which is much more than the originally projected number of 100,000 species. This represents a huge effort by more than 400 contributing specialists throughout Europe and is a unique (standard) reference suitable for many users in science, government, industry, nature conservation and education.

For Neuropterida, data from three Insect orders (Raphidioptera, Megaloptera, Neuroptera), comprising 15 families and 397 species, are included.

## Introduction

In 1998 the European Commission published the European Community Biodiversity Strategy, providing a framework for development of Community policies and instruments in order to comply with the Convention on Biological Diversity. The Strategy recognises the current incomplete state of knowledge at all levels concerning biodiversity, which is a constraint on the successful implementation of the Convention. Fauna Europaea contributes to this Strategy by supporting one of the main themes: to identify and catalogue the components of European biodiversity into a database to serve as a basic tool for science and conservation policies.

With regard to biodiversity in Europe, science and policies depend on knowledge of its components. The assessment of biodiversity, monitoring changes, sustainable exploitation of biodiversity, and much legislative work depend upon a validated overview of taxonomic biodiversity, in which Fauna Europaea plays a major role, providing a web-based information infrastructure with an index of scientific names (including important synonyms) of all living European land and freshwater animals, their geographical distribution at country level and some additional optional information. In this sense the Fauna Europaea database provides a unique reference for many user-groups such as scientists, governments, industries, conservation communities and educational programs.

Fauna Europaea kicked-off in 2000 as an EC-FP5 four years project, delivering its first release in 2004 (Jong et al. 2014). After fourtheen years of steady progress, in order to improve the dissemination of the Fauna Europaea results and to increase the acknowledgement of the Fauna Europaea contributors, novel e-Publishing tools have been applied to prepare data papers of all major taxonomic groups (see below).

Neuropterida is a fairly small group of Insecta, with about 6,500 described species, most of which live in arboreal habitats, but many species are eremial and several live in freshwater habitats. This paper includes a complete list of European taxa of the genus- and family-groups belonging to the Raphidioptera, Megaloptera, and Neuroptera. Recent research suggests that our current appreciation of species diversity of Neuropterida in Europe is still provisional: on the one hand, cryptic, unrecognised taxa are expected to emerge; on the other, the status of some taxa currently treated as one species deserves revisiting. Moreover, a small but constant number of species new for Europe is revealing, mostly on the geographic boundaries of European territory.

Barcoding of European Neuropterida has just begun, one may assume that forthcoming barcode data may lead to various changes of the status of some taxa.

Until the middle of the 20th century the Neuropterida fauna of Europe was rather insufficiently known, and of large regions even largely unknown. The publication of a book on the Neuropterida of Europe (Aspöck et al. 1980) led to a considerable intensification of Neuropterida research in Europe (and adjacent regions) and to a steadily increasing number of publications. Twenty years later another comprehensive review (an annotated catalogue of the Neuropterida of the Western Palaearctic) appeared (Aspöck et al. 2001), which formed the basis for the first version of the catalogue of Neuropterida in Fauna Europaea.

### Data-papers & gap-analysis

In order to improve the dissemination and citation of Fauna Europaea and to increase the acknowledgement of the Fauna Europaea contributors, a special Biodiversity Data Journal Series has been compiled using novel e-Publishing tools, called Contributions on Fauna Europaea, preparing data-papers of all major Fauna Europaea taxonomic groups. This work was initiated during the ViBRANT project and is further supported by the recently started EU BON project. This paper holds the first publication of the Fauna Europaea Neuropterida data sector as a BDJ data paper.

In the EU BON project also further steps will be made on implementing Fauna Europaea as a basic tool and standard reference for biodiversity research in Europe and to evaluate the status of the European taxonomic expertise. The Fauna Europaea data-papers will contribute to a quality assessement on biodiversity data by providing estimates on gaps in taxonomic information and knowledge (see Table 1).

## General description

### Purpose

Fauna Europaea is a database of the scientific names and distribution (at national or -in same cases- regional level) of all living, currently known multicellular European land and fresh-water animal species assembled by a large network of experts, using advanced electronic tools for data collations and validation routines. An extended description of the Fauna Europaea project backgrounds, progress and functioning can be found in Jong et al. 2014. A basic outline is given in the sections below.

**Figure 1.**
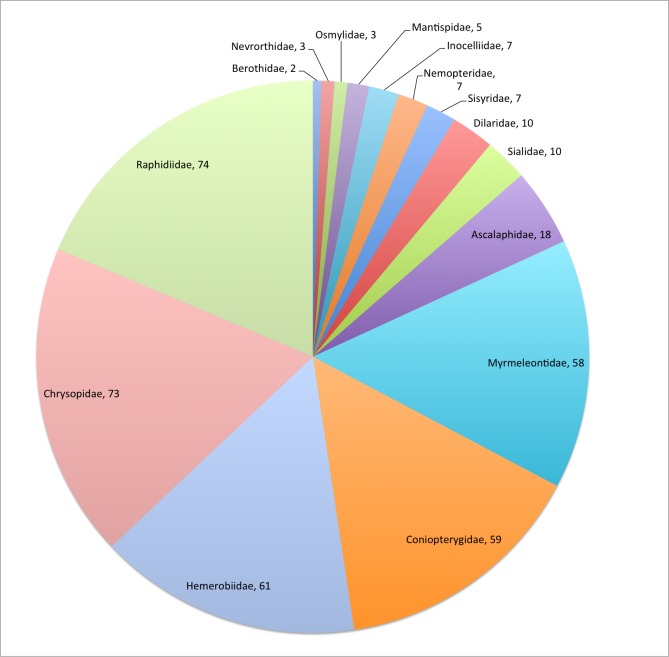
FaEu Neuropterida species per family. See Table 1 for family statistics.

Neuropterida is one of the 58 Fauna Europaea major taxonomic groups, covering 397 species (Fig. 1). The data were collated by a network of 3 specialists (Table 1).

### Additional information

**Figure 2.**
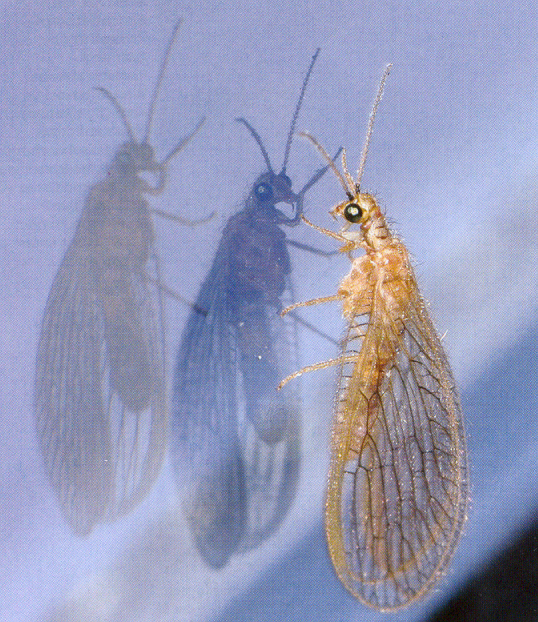
*Nevrorthus apatelios* H. Aspöck, U. Aspöck & Hölzel 1977, photo Peter Sehnal in Aspöck and Aspöck 2010.

The Neuropterida, with about 6,500 described (and possibly 10,000 existing) species, comprise three orders: Raphidioptera, with 241 described valid species in two families; Megaloptera, with about 380 species in two families; and Neuroptera, with at least 6,000 species in 17 families. The small number of species, the heterogeneity of the taxa, the vicariant distribution patterns, and the rich fossil records suggest that the ‘golden age’ of the Neuropterida passed long ago (Aspöck and Aspöck 2007). There are spectacular living fossils among the recent fauna, that is the whole order Raphidioptera, which seems to have hardly changed since the Mesozoic, and, for example, the enigmatic Nevrorthidae among Neuroptera (Fig. 2). Most Neuropterida have terrestrial larvae, only the larvae of Megaloptera and the larvae of neuropteran Nevrorthidae and Sisyridae are truly aquatic (Aspöck 2002).﻿

The basic reference for Neuropterida in Fauna Europaea has been the catalogue of the Western Palaearctic at the beginning of the third millennium (Aspöck et al. 2001). Several European faunal reviews have been published in the following years at a multi-national level (eg. Jedlička et al. 2004), as a national checklist (eg. Letardi et al. 2013), or as a macro area contribution (eg. Letardi et al. 2008, Popov and Letardi 2010), but most of the faunal data published after the above catalogue are scattered in a huge amount of publications. More than 400 papers with faunal data regarding European Neuropterida published between 2000 and 2013 have been analyzed in order to extract potential new reports (Suppl. material 1).

## Project description

### Title

This BDJ data paper includes the taxonomic indexing efforts in Fauna Europaea on European Neuropterida covering the first two versions of Fauna Europaea worked on between 2000 and 2013 (up to version 2.6).

### Personnel

The taxonomic framework of Fauna Europaea includes partner institutes, providing taxonomic expertise and information, and expert networks taking care about data collation.

Every taxonomic group is covered by at least one Group Coordinator responsible for the supervision and integrated input of taxonomic and distributional data of a particular group. For Neuropterida the responsible Group Coordinators are Profs Ulrike & Horst Aspöck and Dr Agostino Letardi.

The Fauna Europaea checklist would not have reached its current level of completion without the input from several groups of specialists. The formal responsibility of collating and delivering the data of relevant families has resided with the below appointed Taxonomic Specialists (see Table 1), while Associate Specialists deserve credit for their important contributions at various levels, including particular geographic regions or (across) taxonomic groups (see Table 2). Details on the Fauna Europaea expert network for Neuropterida can be found here: http://www.faunaeur.org/experts.php?id=681.

Data management tasks are taken care about by the Fauna Europaea project bureau. During the project phase (until 2004) a network of principal partners took care about diverse management tasks: Zoological Museum Amsterdam (general management & system development), Zoological Museum of Copenhagen (data collation), National Museum of Natural History in Paris (data validation) and Museum and Institute of Zoology in Warsaw (NAS extension). Since the formal project ending (2004-2013) all tasks are taken over by the Zoological Museum Amsterdam.

### Study area description

The area study covers the European mainland (Western Palearctic), including the Macaronesian islands, excluding the Caucasus, Turkey, Arabian Peninsula and Northern Africa (see: Geographic coverage).

### Design description

*Standards*. Group coordinators and taxonomic specialists have to deliver the (sub)species names according to strict standards. The names provided by FaEu are scientific names. The taxonomic scope includes issues like, (1) the definition of criteria used to identify the accepted species-group taxa, (2) the hierarchy (classification scheme) for the accommodation of the all accepted species and (3), relevant synonyms, and (4) the correct nomenclature. The Fauna Europaea 'Guidelines for Group Coordinators and Taxonomic Specialists', include the standards, protocols, scope, and limits that provide the instructions for all more then 400 specialists contributing to the project.

**Figure 3.**
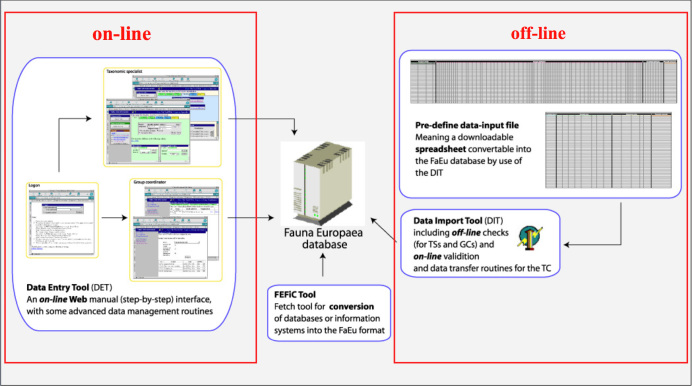
Fauna Europaea on-line (browser interfaces) and off-line (spreadsheets) data entry tools.

*Data management*. The data records could either be entered offline into a preformatted MS-Excel worksheet or directly into the Fauna Europaea transaction database using an online browser interface (see Fig. 3). Since 2013 the data servers are hosted at the Museum für Naturkunde in Berlin.

*Data set*. The Fauna Europaea basic data set consists of: accepted (sub)species names (including authorship), synonyms names (including authorship), taxonomic hierarchy / classification, misapplied names (including misspellings and alternative taxonomic views), homonym annotations, expert details, European distribution (at country level), Global distribution (only for European species), taxonomic reference (optional), occurrence reference (optional).

### Funding

Fauna Europaea was funded by the European Commission under the Fifth Framework Programme and contributed to the Support for Research Infrastructures work programme with Thematic Priority Biodiversity (EVR1-1999-20001) for a period of four years (1 March 2000 - 1 March 2004), including a short 'NAS extension', allowing EU candidate accession countries to participate. Follow-up support was given by the EC-FP5 EuroCAT project (EVR1-CT-2002-20011), by the EC-FP6 ENBI project (EVK2-CT-2002-20020), by the EC-FP6 EDIT project (GCE 018340), by the EC-FP7 PESI project (RI-223806) and by the EC-FP7 ViBRANT project (RI-261532). Continuing management and hosting of the Fauna Europaea services was supported by the University of Amsterdam (Zoological Museum Amsterdam) and SARA/Vancis. Recently the hosting of Fauna Europaea was taken over by the Museum für Naturkunde in Berlin, supported by the EC-FP7 EU BON project (grant agreement №308454).

## Sampling methods

### Study extent

See spatial coverage and geographic coverage descriptions.

### Sampling description

Fauna Europaea data have been assembled by principal taxonomic specialists, based on their individual expertise, Which includes studies of the literature, collection research, and field observations. In total no less than 476 experts contributed taxonomic and/or faunistic information for Fauna Europaea. The vast majority of the experts are from Europe (including EU non-member states). As a unique feature, Fauna Europaea funds were set aside for paying/compensating for the work of taxonomic specialists and group coordinators (around five Euro per species).

To facilitate data transfer and data import, sophisticated on-line (web interfaces) and off-line (spreadsheets) data-entry routines were built, well integrated within an underlying central Fauna Europaea transaction database (see Fig. 3). This included advanced batch data import routines and utilities to display and monitor the data processing within the system. In retrospect, it seems that the off-line submission of data was probably the best for bulk import during the project phase, while the on-line tool was preferred to enter modifications in later versions. This data management system works well until its replacement in 2013.

A first release of the Fauna Europaea index via the web-portal has been presented at 27^th^ of September 2004, the most recent release (version 2.6.2) was launched at 29 August 2013. An overview of Fauna Europaea releases can be found at: http://www.faunaeur.org/about_fauna_versions.php.

### Quality control

Fauna Europaea data are unique in a sense that they are fully expert based. Selecting leading experts for all groups included a principal assurance of the systematic reliability and consistency of the Fauna Europaea data.

Further all Fauna Europaea data sets have been intensively reviewed at regional and thematic validation meetings, at review sessions at taxonomic symposia (for some groups), by Fauna Europaea Focal Points (during the FaEu-NAS and PESI projects) and by various end-users sending annotations using the web form at the web-portal. Additional validation on gaps and correct spelling was effected by the validation office at the MNHN in Paris.

Checks on technical and logical correctness of the data were implemented in the data entry tools, including around "Taxonomic Integrity Rules". This validation tool proved to be of huge value for both the experts and project management, and significantly contribute(d) to preparation of a remarkably clean and consistent data set.

This thorough reviewing makes Fauna Europaea the most scrutinised data sets in its domain. In general, after the initial release, we expected to get taxonomic data for 99.3% of the known European fauna, the faunistic coverage being less complete, but nevertheless holding 90-95% of the total fauna (Jong et al. 2014). Recognised gaps in Neuropterida includes species of several families, but the larger estimate gaps concern Chrysopidae and Coniopterygidae in particular. To our present knowledge, the taxonomic coverage for Neuropterida is near 100% (see Table 1), but the distribution by country is still largely incomplete, especially for the Coniopterygidae.

To optimise the use and implementation of a uniform and correct nomenclature, a cross-referencing of the *Fauna Europaea* Neuropterida data-set with relevant nomenclators and taxonomic catalogues, including Neuropterida Species of the World, is recommended, following the global efforts on establishing a so-called 'Global Names Architecture' (e.g. Pyle and Michel 2008).

### Step description

By evaluating team structure and life cycle procedures (data-entry, validation, updating, etc.), clear definitions of roles of users and user-groups, according to the taxonomic framework were established, including ownership and read and writes privileges, and their changes during the project life-cycle. In addition, guidelines on common data exchange formats and codes have been issued (see also the 'Guidelines' document).

## Geographic coverage

### Description

**Figure 4.**
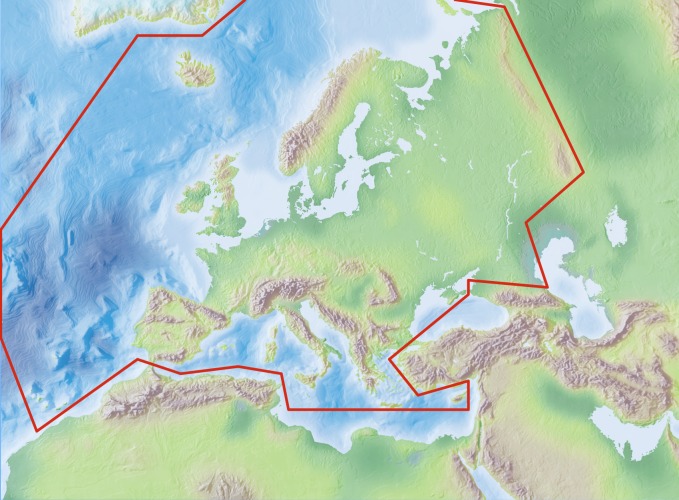
Fauna Europaea geographic coverage ('minimal Europe').

Species and subspecies distributions in Fauna Europaea are registered at least at country level, meaning political countries. For this purpose the FaEu geographical system basically follows the TDWG standards. The covered area includes the European mainland (Western Palearctic), plus the Macaronesian islands (excl. Cape Verde Islands), Cyprus, Franz Josef Land and Novaya Zemlya. Western Kazakhstan and the Caucasus are excluded (see Fig. 4 and coordinates below).

The focus is on species (or subspecies) of European multicellular animals of terrestrial and freshwater environments. Species in brackish waters, occupying the marine/freshwater or marine/terrestrial transition zones, are generally excluded.

The following additional species of Neuropterida have been recorded in Europe since the last version (August 2013):

Ascalaphidae:

*Ascalaphus festivus* (Rambur, 1842) has been found in Sardinia (Pantaleoni et al. 2013).

*Deleproctophylla bleusei* Kimmins, 1949, occurs in the southeast of Spain, has been overlooked (misidentified) so far (Monserrat et al. 2014).

Chrysopidae:

A new species of *Chrysoperla* Steimann, *C. heidarii*, has been described by Henry et al. 2014 from Eastern Aegean islands (Samos, Greece), Lesser Caucasus Mountains (Armenia and Georgia), and northern Iran. A previous synonym of *Chrysopa pallens* Rambur, 1838, has been considered as valid species as *Chrysopa gibeauxi* (Leraut, 1989) by Tillier et al. 2014 and has been found in France, Poland, Finland and North Korea.

Coniopterygidae:

A new species of the genus *Helicoconis* Enderlein, *H. tatrica* (emendation of *H. tatricus*; *Helicoconis* is feminine), has been described by Vidlička (Vidlička 2014) from Slovakia.

Hemerobiidae:

*Hemerobius bolivari* Banks, 1910, a species, widely distributed in South America, has been introduced to Portugal (Monserrat et al. 2013). We do not think that it is really an "invasive" species, but an introduced Neozoon.

Mantispidae:

A new species of the genus *Mantispa* Illiger, *M. incorrupta*, has been described by Monserrat (Monserrat 2014a) from Central Spain.

Myrmeleontidae:

A new species of *Myrmeleon* Linneaus, *M. tschernovi*, has been described by Krivokhatsky (Krivokhatsky et al. 2014) from Kaliningrad Province, Russia. *Nedroledon anatolicus* was found in Macedonia (Kacirek 2013).

Nevrorthidae:

A new species of *Nevrorthus* Costa, *N. reconditus*, has been described by Monserrat & Gavira (Monserrat and Gavira 2014) from the south of Spain.

Sialidae:

Two of the species in Fauna Europaea – *Sialis gonzalezi* Vshivkova, 1985, and *S. dorochovae* Vshivkova, 1985, are confirmed or at least probably synonyms (Aspöck et al. 2001, Monserrat 2014b); two other species – *S. abhasica* Vshivkova, 1985, and *S. klingstedti* Vshivkova, 1985, are possibly valid. Thus, Europe may harbour 8 species of the genus *Sialis* Latreille.

### Coordinates

Mediterranean (N 35°) and Arctic Islands (N 82°) Latitude; Atlantic Ocean (Mid-Atlantic Ridge) (W 30°) and Urals (E 60°) Longitude.

## Taxonomic coverage

### Description

The Fauna Europaea database contains the scientific names of all living European land and freshwater animal species, including numerous infra-groups and synonyms. More details about the conceptual background of Fauna Europaea and standards followed are described in the project description papers.

This data paper covers the Neuropterida content of Fauna Europaea, including 15 families 397 species, 21 subspecies and 12 (sub)species synonyms (see Fig. 1). Higher ranks are given below, the species list can be download (see: Data resources). Some additional notes on the Taxonomic coverage of Neuroptida in Fauna Europaea:

Ascalaphidae: Presently there are 18 species in the list plus 1 subspecies (*Libelloides rhomboideus cretensis*) in addition to the species and nominate subspecies. The current number of species of Ascalaphidae recorded from Europe is (at least) 20, since *Ascalaphus festivus* and *Deleproctophylla bleusei* are not yet in the database.

Chrysopidae: The current in FaEu is 73, however, there is 1 additional (nominal) subspecies: *Pseudomallada flavifrons nigropunctata*.

Raphidiidae: The number of species in the database is 74, in addition there are, however, 7 subspecies (in addition to the nominate subspecies). Of these 7 subspecies 2, namely Raphidia (Raphidia) ophiopsis alcoholica and Raphidia (Raphidia) ophiopsis mediterranea, have received the status of species. Thus, the current number of species of Raphidiidae recorded in Europe (in the sense of FaEu) is 76 plus 5 subspecies.

Sialidae: In the database there are 8 species, 2 of which are proven (*Sialis gonzalezi*) or probable (*Sialis dorochovae*) synonyms. However, 2 possibly valid species (*Sialis abchasica*, *Sialis klingstedti*) have been recorded from European parts of Russia. Thus, the real number of species of Sialidae occurring in Europe may be 8, hardly more.

### Taxa included

**Table:** 

Rank	Scientific Name	Common Name
kingdom	Animalia	
subkingdom	Eumetazoa	
phylum	Arthropoda	
subphylum	Hexapoda	
class	Insecta	
order	Megaloptera	
family	Sialidae	alderfly
genus	*Sialis* Latreille, 1802	
order	Neuroptera	lacewing
suborder	Hemerobiiformia	
family	Berothidae	beaded lacewing
subfamily	Berothinae	
genus	*Isoscelipteron* Costa, 1863	
family	Chrysopidae	green lacewing
subfamily	Chrysopinae	
tribe	Belonopterygini	
genus	*Italochrysa* Principi, 1946	
tribe	Chrysopini	
genus	*Atlantochrysa* Hölzel, 1970	
genus	*Brinckochrysa* Tjeder, 1966	
genus	*Chrysopa* Leach in Brewster, 1815	
genus	*Chrysoperla* Steinmann, 1964	
genus	*Chrysotropia* Navás, 1911	
genus	*Cunctochrysa* Hölzel, 1970	
genus	*Nineta* Navás, 1912	
genus	*Peyerimhoffina* Lacroix, 1920	
genus	*Pseudomallada* Tsukaguchi, 1995	
genus	*Rexa* Navás, 1919	
genus	*Suarius* Navás, 1914	
subfamily	Nothochrysinae	
genus	*Hypochrysa* Hagen, 1866	
genus	*Nothochrysa* McLachlan, 1868	
family	Coniopterygidae	dustywing
subfamily	Aleuropteryginae	
tribe	Aleuropterygini	
genus	*Aleuropteryx* Löw, 1885	
tribe	Conwentziini	
genus	*Conwentzia* Enderlein, 1905	
genus	*Hemisemidalis* Meinander, 1972	
genus	*Semidalis* Enderlein, 1905	
tribe	Fontenelleini	
genus	*Helicoconis* Enderlein, 1905	
genus	*Vartiana* H. Aspöck & U. Aspöck, 1965	
subfamily	Coniopteryginae	
tribe	Coniopterygini	
genus	*Coniopteryx* Curtis, 1834	
genus	*Nimboa* Navás, 1915	
genus	*Parasemidalis* Enderlein, 1905	
family	Dilaridae	pleasing lacewing
subfamily	Dilarinae	
genus	*Dilar* Rambur, 1838	
family	Hemerobiidae	brown lacewing
subfamily	Drepanepteryginae	
genus	*Drepanepteryx* Leach in Brewster, 1815	
subfamily	Hemerobiinae	
genus	*Hemerobius* Linnaeus, 1758	
genus	*Wesmaelius* Krüger, 1922	
subfamily	Megalominae	
genus	*Megalomus* Rambur, 1842	
subfamily	Microminae	
genus	*Micromus* Rambur, 1842	
subfamily	Notiobiellinae	
genus	*Psectra* Hagen, 1866	
subfamily	Sympherobiinae	
genus	*Sympherobius* Banks, 1904	
family	Mantispidae	mantisfly
subfamily	Mantispinae	
genus	*Mantispa* Illiger in Kugelann, 1798	
genus	*Nampista* Navás, 1914	
family	Osmylidae	
subfamily	Osmylinae	
genus	*Osmylus* Latreille, 1802	
family	Sisyridae	spongilla fly
genus	*Sisyra* Burmeister, 1839	
suborder	Myrmeleontiformia	
family	Ascalaphidae	owlfly
subfamily	Ascalaphinae	
genus	*Bubopsis* McLachlan, 1898	
genus	*Deleproctophylla* Lefèbvre, 1842	
genus	*Libelloides* Schäffer, 1763	
genus	*Puer* Lefèbvre, 1842	
family	Myrmeleontidae	antlion
subfamily	Myrmeleontinae	
tribe	Acanthaclisini	
genus	*Acanthaclisis* Rambur, 1842	
genus	*Synclisis* Rambur, 1842	
tribe	Dendroleontini	
genus	*Dendroleon* Brauer, 1866	
genus	*Tricholeon* Esben-Petersen, 1925	
tribe	Glenurini	
genus	*Gymnocnemia* Schneider, 1845	
genus	*Megistopus* Rambur, 1842	
genus	*Nedroledon* Navás, 1914	
tribe	Myrmecaelurini	
genus	*Aspoeckiana* Hölzel, 1969	
genus	*Lopezus* Navás, 1913	
genus	*Myrmecaelurus* Costa, 1855	
genus	*Nohoveus* Navás, 1919	
genus	*Solter* Navás, 1912	
tribe	Myrmeleontini	
genus	*Euroleon* Esben-Petersen, 1918	
genus	*Myrmeleon* Linnaeus, 1767	
tribe	Nemoleontini	
genus	*Creoleon* Tillyard, 1918	
genus	*Delfimeus* Navás, 1912	
genus	*Deutoleon* Navás, 1927	
genus	*Distoleon* Banks, 1810	
genus	*Macronemurus* Costa, 1855	
genus	*Nemoleon* Navás, 1909	
genus	*Neuroleon* Navás, 1909	
genus	*Noaleon* Holzel 1972	
tribe	Nesoleontini	
genus	*Cueta* Navás, 1911	
subfamily	Palparinae	
tribe	Palparini	
genus	*Palpares* Rambur, 1842	
family	Nemopteridae	spoonwing
subfamily	Crocinae	
genus	*Josandreva* Navás, 1906	
genus	*Pterocroce* Withycombe, 1923	
subfamily	Nemopterinae	
genus	*Lertha* Navás, 1910	
genus	*Nemoptera* Latreille, 1802	
suborder	Nevrorthiformia	
family	Nevrorthidae	
genus	*Nevrorthus* Costa, 1863	
order	Raphidioptera	snakefly
family	Inocelliidae	
genus	*Fibla* Navás, 1915	
genus	*Inocellia* Schneider, 1843	
genus	*Parainocellia* H. Aspöck & U. Aspöck, 1968	
family	Raphidiidae	
genus	*Atlantoraphidia* H. Aspöck & U. Aspöck, 1968	
genus	*Calabroraphidia* Rausch, H. Aspöck & U. Aspöck, 2004	
genus	*Dichrostigma* Navás, 1909	
genus	*Harraphidia* Steinmann, 1963	
genus	*Hispanoraphidia* H. Aspöck & U. Aspöck, 1968	
genus	*Italoraphidia* H. Aspöck & U. Aspöck, 1968	
genus	*Ohmella* H. Aspöck & U. Aspöck, 1968	
genus	*Ornatoraphidia* H. Aspöck & U. Aspöck, 1968	
genus	*Parvoraphidia* H. Aspöck & U. Aspöck, 1968	
genus	*Phaeostigma* Navás, 1909	
genus	*Puncha* Navás, 1915	
genus	*Raphidia* Linnaeus, 1758	
genus	*Subilla* Navás, 1916	
genus	*Tjederiraphidia* H. Aspöck, U. Aspöck & Rausch, 1985	
genus	*Turcoraphidia* H. Aspöck & U. Aspöck, 1968	
genus	*Ulrike* H. Aspöck, 1968	
genus	*Venustoraphidia* H. Aspöck & U. Aspöck, 1968	
genus	*Xanthostigma* Navás, 1909	

## Temporal coverage

**Living time period:** Currently living.

### Notes

Currently living multicellular, terrestrial and freshwater animals in stable populations, largely excluding (1) rare / irregular immigrants, (2) alien / invasive species, (3) accidental or deliberate releases of exotic (pet)species, (4) domesticated animals, (5) non-native species imported and released for bio-control or (6) non-native species largely confined to hothouses.

## Usage rights

### Use license

Open Data Commons Attribution License

### IP rights notes

Fauna Europaea data are licensed under CC BY SA version 4.0. The property rights of experts over their data is covered under the SMEBD conditions. For more copyrights and citation details see: http://www.faunaeur.org/copyright.php

## Data resources

### Data package title

Fauna Europaea - Neuropterida

### Resource link

http://www.faunaeur.org/Data_papers/FaEu_Neuropterida_2.6.2.zip

### Alternative identifiers

http://www.faunaeur.org/experts.php?referrer=experts_search&id=681

### Number of data sets

2

### Data set 1.

#### Data set name

Fauna Europaea - Neuropterida version 2.6.2 - species

#### Data format

CSV

#### Number of columns

25

#### Character set

UTF-8

#### Download URL

http://www.faunaeur.org/Data_papers/FaEu_Neuropterida_2.6.2.zip

#### Description

**Data set 1.** 

Column label	Column description
datasetName	The name identifying the data set from which the record was derived (http://rs.tdwg.org/dwc/terms/datasetName).
version	Release version of data set
versionIssued	Issue data of data set version.
rights	Information about rights held in and over the resource (http://purl.org/dc/terms/rights).
rightsHolder	A person or organization owning or managing rights over the resource (http://purl.org/dc/terms/rightsHolder).
accessRights	Information about who can access the resource or an indication of its security status (http://purl.org/dc/terms/accessRights).
taxonID	An identifier for the set of taxon information (http://rs.tdwg.org/dwc/terms/taxonID)
parentNameUsageID	An identifier for the name usage of the direct parent taxon (in a classification) of the most specific element of the scientificName (http://rs.tdwg.org/dwc/terms/parentNameUsageID).
scientificName	The full scientific name, with authorship and date information if known (http://rs.tdwg.org/dwc/terms/scientificName).
acceptedNameUsage	The full name, with authorship and date information if known, of the currently valid (zoological) taxon (http://rs.tdwg.org/dwc/terms/acceptedNameUsage).
originalNameUsage	The original combination (genus and species group names), as firstly established under the rules of the associated nomenclaturalCode (http://rs.tdwg.org/dwc/terms/originalNameUsage).
family	The full scientific name of the family in which the taxon is classified (http://rs.tdwg.org/dwc/terms/family).
familyNameId	An identifier for the family name.
genus	The full scientific name of the genus in which the taxon is classified (http://rs.tdwg.org/dwc/terms/genus).
subgenus	The full scientific name of the subgenus in which the taxon is classified. Values include the genus to avoid homonym confusion (http://rs.tdwg.org/dwc/terms/subgenus).
specificEpithet	The name of the first or species epithet of the scientificName (http://rs.tdwg.org/dwc/terms/specificEpithet).
infraspecificEpithet	The name of the lowest or terminal infraspecific epithet of the scientificName, excluding any rank designation (http://rs.tdwg.org/dwc/terms/infraspecificEpithet).
taxonRank	The taxonomic rank of the most specific name in the scientificName (http://rs.tdwg.org/dwc/terms/infraspecificEpithet).
scientificNameAuthorship	The authorship information for the scientificName formatted according to the conventions of the applicable nomenclaturalCode (http://rs.tdwg.org/dwc/terms/scientificNameAuthorship).
authorName	Author name information
namePublishedInYear	The four-digit year in which the scientificName was published (http://rs.tdwg.org/dwc/terms/namePublishedInYear).
Brackets	Annotation if authorship should be put between parentheses.
nomenclaturalCode	The nomenclatural code under which the scientificName is constructed (http://rs.tdwg.org/dwc/terms/nomenclaturalCode).
taxonomicStatus	The status of the use of the scientificName as a label for a taxon (http://rs.tdwg.org/dwc/terms/taxonomicStatus).
resourceDescription	An account of the resource, including a data-paper DOI (http://purl.org/dc/terms/description)

### Data set 2.

#### Data set name

Fauna Europaea - Neuropterida version 2.6.2 - hierarchy

#### Data format

CSV

#### Number of columns

12

#### Character set

UTF-8

#### Download URL

http://www.faunaeur.org/Data_papers/FaEu_Neuropterida_2.6.2.zip

#### Description

**Data set 2.** 

Column label	Column description
datasetName	The name identifying the data set from which the record was derived (http://rs.tdwg.org/dwc/terms/datasetName).
version	Release version of data set.
versionIssued	Issue data of data set version.
rights	Information about rights held in and over the resource (http://purl.org/dc/terms/rights).
rightsHolder	A person or organization owning or managing rights over the resource (http://purl.org/dc/terms/rightsHolder).
accessRights	Information about who can access the resource or an indication of its security status (http://purl.org/dc/terms/accessRights).
taxonName	The full scientific name of the higher-level taxon
scientificNameAuthorship	The authorship information for the scientificName formatted according to the conventions of the applicable nomenclaturalCode (http://rs.tdwg.org/dwc/terms/scientificNameAuthorship).
taxonRank	The taxonomic rank of the most specific name in the scientificName (http://rs.tdwg.org/dwc/terms/infraspecificEpithet).
taxonID	An identifier for the set of taxon information (http://rs.tdwg.org/dwc/terms/taxonID)
parentNameUsageID	An identifier for the name usage of the direct parent taxon (in a classification) of the most specific element of the scientificName (http://rs.tdwg.org/dwc/terms/parentNameUsageID).
resourceDescription	An account of the resource, including a data-paper DOI (http://purl.org/dc/terms/description)

## Supplementary Material

Supplementary material 1 references 2000-2013 Data type: bibliography
Brief description: data base of papers used to update Neuroperida in Fauna Europaea vers. 2.6.2
File: oo_32493.doc Letardi A.

## Figures and Tables

**Table 1.** Taxonomic specialists per family in Neuropterida and their responsibilities. The actual numbers of databased species are given per family. For most families is also given an indication of the actual number of known/described species (showing a potential information gap) plus an estimate of the total number of existing species (i.e., described/known plus undescribed/undiscovered) for Europe.

**TAXONOMY**		**EUROPE**		
**FAMILY**	**SPECIALIST(S)**	**DATABASED SPECIES (Fauna Europaea)**	**TOTAL DESCRIBED SPECIES (information-gap)**	**TOTAL ESTIMATED SPECIES (knowledge-gap)**
Ascalaphidae	Profs Ulrike Aspöck & Horst Aspöck and Dr Agostino Letardi	18	20	~20
Berothidae	Profs Ulrike Aspöck & Horst Aspöck and Dr Agostino Letardi	2	2	~2
Chrysopidae	Profs Ulrike Aspöck & Horst Aspöck and Dr Agostino Letardi	73	74	~80
Coniopterygidae	Profs Ulrike Aspöck & Horst Aspöck and Dr Agostino Letardi	59	•	~75
Dilaridae	Profs Ulrike Aspöck & Horst Aspöck and Dr Agostino Letardi	10	•	~12
Hemerobiidae	Profs Ulrike Aspöck & Horst Aspöck and Dr Agostino Letardi	61	62	~70
Inocelliidae	Profs Ulrike Aspöck & Horst Aspöck and Dr Agostino Letardi	7	7	~7
Mantispidae	Profs Ulrike Aspöck & Horst Aspöck and Dr Agostino Letardi	5	6	~6
Myrmeleontidae	Profs Ulrike Aspöck & Horst Aspöck and Dr Agostino Letardi	58	60	~70
Nemopteridae	Profs Ulrike Aspöck & Horst Aspöck and Dr Agostino Letardi	7	7	~7
Nevrorthidae	Profs Ulrike Aspöck & Horst Aspöck and Dr Agostino Letardi	3	4	•
Osmylidae	Profs Ulrike Aspöck & Horst Aspöck and Dr Agostino Letardi	3	3	~3
Raphidiidae	Profs Ulrike Aspöck & Horst Aspöck and Dr Agostino Letardi	74	76	~80
Sialidae	Profs Ulrike Aspöck & Horst Aspöck and Dr Agostino Letardi	10	6	~8
Sisyridae	Profs Ulrike Aspöck & Horst Aspöck and Dr Agostino Letardi	7	7	~7
