# Supplementary material for: Fauna Europaea: Neuropterida (Raphidioptera, Megaloptera, Neuroptera)
Source: Biodivers Data J. 2015 Apr 17;(3):e4830. doi: 10.3897/BDJ.3.e4830 (PMC4411496; doi:10.3897/BDJ.3.e4830)
Supplement: Supplementary material 1 — references 2000-2013 [file biodiversity_data_journal-3-e4830-s001.doc]

A.A.V.V., 2005. L’actualisation de l’inventaire des Zones Naturelles d’Intérêt Ecologique, Faunistique et Floristique de Provence Alpes Côte d’Azur : ANNEXE 1 : Listes des espèces et habitats déterminants et remarquables. DIREN PACA, juillet 2005, 55 pp.

Aberlenc, H.-P. 2008. Les insectes du bois de Païolive: premier supplemént à l’inventaire. Le Cahiers de Païolive 1: 155-167.

Aberlenc, H.-P.; Lentenois, P. 2003. Les insectes du bois de Païolive: 55-72. In : Holthof, J.F.; Schnetzler, J. (Eds), De Saint-Eugène en Païolive, Montmélian et les Vans, La Fontaine de Siloé et Saint-Eugène en Païolive: 320 p.

Abrahám, L. 2000. Alderfly (Megaloptera) and lacewing (Neuroptera) fauna of the Villány Hills, South Hungary. Dunántúli Dolgozatok (A) Természettudományi Sorozat [=Studia Pannonica (A) Series Historico-Naturalis] 10:249-266.

Abrahám, L. 2001. Somogy megye recésszárnyú-alkatúinak katalógusa (Megaloptera, Raphidioptera, Neuroptera). Natura Somogyiensis 1:253-260.

Abrahám, L. 2003. A Látrányi Puszta Természetvédelmi Terület recésszárnyú-alkatú (Megaloptera, Raphidioptera, Neuroptera) faunája. Natura Somogyiensis 5: 193-200.

Abrahám, L.; Markó, V.; Vas, J. 2003. Investigations on a neuropteroid community by using different methods. Acta Phytopathologica et Entomologica Hungarica 38:199-207.

Acevedo, F.; Monserrat, V. J.; Badano, D. 2013. Comparative description of larvae of the European species of Distoleon Banks: D. annulatus (Klug, 1834) and D. tetragrammicus (Fabricius, 1798) (Neuroptera, Myrmeleontidae). Zootaxa 3721(5): 488-494.

Ahlén, I. (ed.) 2007. Faunan och floran pa Krusenberg i Uppland. Centrum för biologisk mangfald CBM:s skriftserie14: 170 pp.

Aistleitner, E. 2007. Zur taxonomie und chorologie des schmetterlingshaftes Libelloides lacteus (Brullé, 1832)(Neuroptera, Ascalaphidae). Entomofauna 28(26) :357-368.

Aistleitner, E.; Hölzel+ H. 2012. Zur Kenntnis der Schmetterlingshafte, Florfliegen und Ameisenjungfern (Neuropterida: Ascalaphidae, Chrysopidae, Myrmeleonidae) der Kapverden (Cabo Verde). Zeitschruft der Arbeitsgemeinschaft Österreichischer Entomologe, 64:119-124.

Aistleitner, U.; Gruppe, A. 2004. Vorläufige Checkliste der Netzflügler (Insecta: Neruopterida) Vorarlbergs / Austria occ. Vorarlberger Naturschau Forschen und Entdecken 14:153-160.

Alhmedi, A.; Francis, F.; Bodson, B.; Haubruge, E. 2006. Etude de la diversità des pucerons et des auxiliaires aphidiphages relative à la presence d’orties en bordure de champs. Note faunistique de Gembloux 59(2) : 121-124.

Alhmedi, A.; Francis, F.; Bodson, B.; Haubruge, E. 2007. Evaluation de la diversité des pucerons et de leurs ennemis naturels en grandes cultures à proximité de parcelles d’orties. Note faunistique de Gembloux 60(4) : 147-152.

Allemand, R.; Vincent, R. 2000. Compte rendu faunistique del’excursion de la Societe entomologique de France dans le Maconnais (19-21 juin 1999). Bull. Mens. Soc. Linn. Lyon 69(5):85-112.

Alvis, L.; Garcia-Mari, F. 2002. Identification and abundance of Neuropteran species associated with citrus orchads in Valencia, Spain. IOBC/WPRS Citrus Working Group Integrated Control in citrus fruit crops, Valencia, 6-8 November 2002: 18.

Alziar, G.; Lemaire, J.-M. 2012. Les missions entomologiques du Museum d’Histoire Naturelle de Nice (France), 2004-2010. Biocosme Mesogeen 29(3-4): 57-104.

Anderle, F. 2005. Die Neuropterida des Eichkogels bei Mödling (Niederösterreich). Nachrichten der Deutsche Gesellschaft für allgemeine und angewandte Entomologie e.V. 19(3):140-141.

Anderle, F.; Aspöck, U. 2007. Neuropterida (Insecta Endopterygota) of the Nature Reserve Eichkogel (Lower Austria): arguments for protecting an insular biocoenosis in the South of Vienna. Ann. Mus. civ. St. nat. Ferrara, 8: 139-144.

Aspöck, H. 2002. Die Neuropterida der Westpaläarktis. Galathea 13 suppl.: 11-12.

Aspöck, H.; Aspöck, U. 2005. Die Raphidiopteren der Apenninen-Halbinsel: eine biogeographische Analyse. Nachrichten der Deutsche Gesellschaft für allgemeine und angewandte Entomologie e.V. 19(3):142-144.

Aspöck, H.; Aspöck, U. 2007. The Raphidioptera of the Apennines Peninsula: a biogeographical analysis. Ann. Mus. civ. St. nat. Ferrara, 8: 95-106.

Aspöck, H.; Aspöck, U. 2008. Fanghaft, Schmetterlingshaft und Ameisenlöwen auf mitteleuropäischen trockenrasen. In: Wiesbauer, H. 2008. Die Steppe lebt. Felssteppen und trockenrasen in Niederösterreich. St. Pölten: 153-158.

Aspöck, H.; Aspöck, U. 2009. Raphidioptera – Kamelhalsfliegen ein überblick zum einstieg. Entomologica Austriaca 16: 53-72.

Aspöck, H.; Aspöck, U. 2009. Neuropterida (Netzflügler). Pp. 625-627. In: Rabitsch, W.; Essl, F. (eds) 2009. Endemiten. Kostbarkeiten in Österreichs Pflanzen- und Tierwelt. Naturwissenschaftlicher Verein für Kärnten und Umweltbundesamt GmbH, Klagenfurt und Wien, 924 pp.

Aspöck, H.; Aspöck, U. 2009. Die frühe Geschichte der Erforschung der Neuropteren-Familie Coniopterygidae (Insecta: Neuropterida). – In Ch. Kropf & P. Horak (Eds.): Towards a Natural History of Arthropods and Other Organisms. In Memoriam Konrad Thaler. Contributions to Natural History. Scientific papers from the Natural History Museum Bern 12 (fascicule 1): 71-125.

Aspöck, H.; Aspöck, U. 2012. Turcoraphidia amara (Aspöck & Aspöck, 1964) - ein Juwel der Insektenfauna Rumäniens. Buletin de Informare Entomologică 21: 33-35.

Aspöck, H.; Hölzel, H.; Aspöck, U. 2001. Kommentierter Katalog der Neuropterida (Insecta: Raphidioptera, Megaloptera, Neuroptera) der Westpaläarktis. Denisia 2:1-606.

Aspöck, U.; Aspöck, H. 2005. Verbreitungsgrenzen von Neuropterida in Mitteleuropa. Linzer biol. Beitr., 37(1):29-38.

Aspöck, U., Aspöck, H. 2009. Raphidioptera (Snakeflies). In: V.H. Resh & R.T. Cardé (eds.): Encyclopedia of Insects. Second Edition. Academic Press, Elsevier Amsterdam etc.: 864-866.

Aspöck, U.; Aspöck, H. 2013. Nevrorthidae, Sisyridae, Coniopterygidae ... – Der Streit um die niederen Ränge im Stammbaum der Neuroptera. Nachrichten der Deutsche Gesellschaft für allgemeine und angewandte Entomologie e.V. 27:19-20.

Audisio, P. 2002. Litorali sabbiosi e organismi animali. In: A.A.V.V. 2002. Dune e spiagge sabbiose. Ambienti fra terra e mare. Quaderni Habitat, 4: 63-117.

Badano, D. 2008. Contributo alla conoscenza dei Neurotteri della Liguria (Insecta, Neuropterida). Annali del Museo Civico di Storia Naturale “G. Doria” 99: 535-548.

Badano, D. 2010. Sympherobius riudori nuovo per l’Italia (Neuroptera Hemerobiidae). Boll. Soc. entomol. Ital., 142(1):7-9.

Badano, D. 2011. I Neurotteri (Neuropterida) della Liguria. Atti XXIII Congresso Nazionale Italiano di Entomologia, Genova 13-16 Giugno 2011; 54.

Badano, D.; Almeida, J.; Letardi, A. 2011. Redescoberta de Megaloptera em Portugal Continental após um século, uma ordem “renascida” para o país. Arquivos Entomolóxicos 5:53-54.

Badano, D.; Letardi, A. 2010. A review of the Neuropterida of Liguria (North-West Italy). In: Devetak, D.; Lipovsek, S.; Arnett, A.E. (eds) 2010. Proceedings of the 10th Int. Symp. on Neuropterology. Maribor, Slovenia: 83-87.

Badmin, J. 2007. The hemerobiid Drepanepteryx phalaenoides (L.), east Kent. British Journal of Entomology and Natural History 20(3): I-II.

Barclay, M. V. L. 2005. An interesting insect assemblage reared from the bracket fungus Inonotus hispidus (Bull. Ex Fr.) Karst from Hyde Park, Middlesex. British Journal of Entomology and Natural History 18: 41-44.

Barnard, P.C. 2003. Benhs indoor meetings. 14 january 2003. Br. J. Ent. Nat. Hist., 16:196.

Barnard, P.C. 2011. The Royal Entomological Society book of British Insects. Wiley-Blackwell Pub., 383 pp.

Bellstedt, R. 2000. Zur Limnofauna von Nesselbach und Dürrer Floh bei Schnellbach im Thüringer Wald (Landkreis Schmalkalden-Meiningen). Thüringer Faunistische Abhandlungen 7:89-99.

Bellstedt, R.; Schuster, C. 2007. Zur Fauna der „Armen Jacke“ – eines Halbtrockenrasens an der nordabdachung der Fahner Höne (Landkreis Gotha, Thüringen). Thüringer Faunistische Abhandlungen 12:53-67.

Bergsten, J. ; Ekerholm, P. ; Hellqvist, S. ; Hilszczanski, J. ; Nilsson, A. ; Pettersson, R. ; Werner, T. 2003. Insekter och spindeldjur fran Romelsön. Natur i Norr, Umea 22(2): 65-87.

Blaik, T. 2008. New data and remarks on the occurrence of Micromus lanosus (Zeleny, 1962) (Neuroptera: Hemerobiidae) in Poland. Opole Sci. Soc. Natur. J., 41: 49:52.

Blaik, T.; Dobosz, R. 2010. Lacewings (Neuroptera) of the Polish Baltic coast with remarks on Wesmaelius (Kimminsia) balticus (Tjeder, 1931) – a new species of Hemerobiidae to the fauna of Poland. In: Devetak, D.; Lipovsek, S.; Arnett, A.E. (eds) 2010. Proceedings of the 10th Int. Symp. on Neuropterology. Maribor, Slovenia: 97-112.

Bleckmann, C.; Lienenbecker, H. 2005. Eine kolonie des Ameisenlöwen (Euroleon nostra) in einer wohnsiedlung in Steinhagen/Kreis Gütersloh. Berichte des Naturwissenschaftliche Verein für Bielefeld 45: 279-284.

Bo, T.; Fenoglio, S. 2005. Sulla presenza di alcuni macroinvertebrati bentonici rari o interessanti nei torrenti e fiumi dell’appennino piemontese. Rivista Piemontese di Storia Naturale 26:123-128.

Boemo, B.; Battistella, S.; Colla, A. 2007. Contributo alla conoscenza degli artropodi di una collina in flysch (M.te Fiascone = M.te Valerio) alla periferia di Trieste (Italia, Friuli Venezia Giulia). Atti del Museo civico di Storia Naturale di Trieste 53: 25-70.

Borysenko, N. N. 2013. Contribution to Neuroptera of north-eastern part of Cherchassy region. Ukrainska Entomofaunistyka 4(1): 43-47.

Bottelli, F.; Calonghi, E.; Daccordi, M.; Giachino, P. M. 2009. Catalogo della Collezione Entomologica “Agostino Dodero” in Oropa, (Biella, Italia), 83-116. In: Bottelli F. & Giachino P. M. (eds), 2009 Contributo alle conoscenze naturalistiche della Valle Oropa (Biella, Italia). Memorie Associazione Naturalistica Piemontese, 11: 120 pp

Bozsik A. 2003. The lacewing fauna (Neuroptera: Chrysopidae) of Debrecen. Proceedings of 3rd International Plant Protection Symposium, Debrecen University, 15-16 October 2003, Debrecen: 141.

Bozsik A.; Gonzalez Ruiz, R. 2006. First data on the sibling species of the common green lacewings in Spain (Neuroptera: Chrysopidae). Presented at-4th International Plant Protection Symposium. Debrecen, Hungary, 18-19 October 2006.

Bozsik, A. ; Mignon, J.; Gaspar, C. 2000. Contribution a la connaissance des Chrysopidae de Belgique: bilan des captures realisées a Gembloux. Notes fauniques de Gembloux 41:3-10.

Bozsik, A. ; Mignon, J.; Gaspar, C. 2002. The green lacewings in Belgium (Neuroptera: Chrysopidae). Acta Zoologica Academiae Scientiarum Hungaricae 48 (Suppl. 2), 53-59.

Brandmayr, P.; Gangale, C.; Mazzei, A.; Mingozzi, A.; Pizzolotto, R.; Urso, S.; Scalercio, S.; Tripepi, S. Aloise, G.; Ouzunov, D. 2013. L’approfondimento: la biodiversità animale e vegetale della Sila. Sinergie, rapporti di ricerca 37: 71-93.

Brettfeld, R.; Bellstedt, R. 2003. Zur Limnofauna des Bergbaches Schweina im Wartburgkreis. Thüringer Faunistische Abhandlungen 9:61-72.

Brûlè, S.; Touroult, J.; Dalens,P.-H. (cood.) 2011. Résultats de l’inventaire entomologique du site de Saut Pararé, reserve des Nouragues (Guyane), 2009-2010. Rapport de la Société entomologique Antilles-Guyane, SEAG, ONF, 120pp.

Campanaro, A.; Hardersen, S.; Mason, F. (eds) 2007. Piano di gestione della Riserva Naturale Statale e Sito Natura 2000 “Bosco della Fontana”. Quaderni Conservazione Habitat 4: 1-221.

Canard, M.. 2001. Présence en Grèce continentale de Chrysoperla carnea (Stephens, 1836) sensu stricto (Neuropt., Chrysopidae). Bulletin de la Société Entomologique de France 106:416.

Canard, M. 2003. Chrysoperla mutata (McLachlan, 1898), une espèce nouvelle pour la Crète et l’ile de Chios, Grèce (Neuropt., Chrysopidae). Bulletin de la Société Entomologique de France 108: :216.

Canard, M. 2004. World distribution of the genus Nineta Navas 1912 (Neuroptera: Chrysopidae), with some taxonomic notes. Denisia 13: 153-161. In: A.A.V.V. 2004. Entomologie und Parasitologie - Festschrift zum 65. Geburtstag von Horst Aspöck, Wissenschaftliche Redaktion: Ulrike Aspock, Denisia 13: 635 pp.

Canard, M. 2007. Deux Chrysopes nouvelles pour la faune de Chypre : Italochrysa italiaca (Rossi, 1790) et Cunctochrysa baetica (Hölzel, 1972) (Neur., Chrysopidae). Bulletin de la Société Entomologique de France 112(3):406.

Canard, M.; Giacomino, M.; Jacquemin, G.; Thierry, D.; Tillier, P.; Villenave-Chasset, J. 2009. Complements a la cartographie des Chrysopes en France (Neuroptera, Chrysopidae). R.A.R.E., 18(1) : 70-73.

Canard, M.; Giacomino, M.; Thierry, D.; Tillier, P.; Villenave-Chasset, J. 2011. Deuxième complément à la cartographie dés Chrysopes en France (Neuroptera, Chrysopidae). R.A.R.E., 20(2) : 45-52.

Canard, M.; Jacquemin, G. 2006. Capture en France d’une Chrysope rare ou méconnue: Chrysopa dubitans McLachlan, 1887 (Neuroptera). Bull. Soc. Entomol. France, 111(4): 483-485.

Canard, M.; Jacquemin, G. 2013. Présence de Pseudomallada inornatus (Navás) dans le nord-est de la France (Neuroptera, Chrysopidae). Bulletin de la Société entomologique de France 118(4): 489-492.

Canard, M.; Letardi, A.; Thierry, D. 2007. The rare Chrysopidae (Neuroptera) of southwestern Europe. Acta Oecologica 31(3): 290-298.

Canard, M.; Mazel, R.; Thierry, D. 2006. Repartition des Chrysopes en France (Neuroptera, Chrysopidae). Bull. Soc. Entomol. France, 111(3): 353-366.

Canard, M.; Mazel, R.; Tillier, P.; Danfloss, S.; Thierry, D. 2007. Cartographie des Chrysopes en France (Neuroptera, Chrysopidae). R.A.R.E., 16(1) : 9-21.

Canard, M.; Thierry, D.; Whittington, A.E.; Bozsik, A. 2010. The actual annual occurrence of the green lacewings of northwestern Europe (Neuroptera: Chrysopidae). In: Devetak, D.; Lipovsek, S.; Arnett, A.E. (eds) 2010. Proceedings of the 10th Int. Symp. on Neuropterology. Maribor, Slovenia: 127-134.

Carotti, G. 2010. Neurotteroidei e Mecotteri della Riserva Naturale Montagna di Torricchio (Marche, Italia Centrale)(Raphidioptera, Neuroptera, Mecoptera). La Riserva naturale di Torricchio 13: 53-63.

Carotti, G.; Nicoli Aldini, R.; Letardi, A. 2009. Insecta Neuropterida delle Marche: attuale stato delle conoscenze. Proceedings del XXII Congresso Nazionale Italiano di Entomologia, Ancona, 15-18 Giugno 2009: 62.

Carrieres, E. 2001. Revision and additions to the list of lacewings (Megaloptera, Neuroptera, Raphidioptera) and scorpion flies (Mecoptera) of Luxembourg. Bull. Soc. Nat. luxemb. 102: 91-96.

Carrieres, E.; Meyer, M. 2003. Catalogage et révision des collections sèches du Musée national d’histoire naturelle: Neuropteroidea (Raphidioptera, Megaloptera, Plannipennia) et Mecoptera. Bull. Soc. Nat. luxemb., 104: 89-94.

Cartier, G. 2000. Addenda aux insectes de Rueil Malmaison. Determinations posterieures au 14 Septembre 1999. Entomologiste 56(4): 161-165.

Ceccolini, F.; Terzani, F. 2012. Presenza di Chrysotropia ciliata (Wesmael, 1841) in Toscana (Neuroptera: Chrysopidae). Onychium 9: 105-107.

Cesaroni, C. 2007. Neurotterofauna della Sardegna. Tesi di Dottorato 2006-2007 Università degli Studi di Sassari, 184 pp.

Chapman, J.W.; Reynolds, D.R.; Smith, A.D.; Smith, E.T.; Woiwod, I.P. 2004. An aerial netting study of insects migrating at high altitude over England. Bulletin of entomological research 94(2): 123-136.

Chireceanu, C. 2007. Species diversity of insects in pear ecosystem in Băneasa-Bucharest. Entomol. Rom., 12: 243-249.

Cianferoni, F.; Fabiano, F.; Mazza, G.; Rocchi, S.; Terzani, F.; Zinetti, F. 2009. Gli invertebrati della Riserva Naturale Integrale di Sasso Fratino. In: Bottacci, A. (eds) 2009. La Riserva Naturale Integrale di Sasso Fratino: 1959-2009. 50 anni di conservazione della biodiversità. CFS/UTB Pratovecchio: 227-248.

Cocquemot, C.; Rungs, C. 2009. Listes des arthropodes terrestres recenses dans les reserves naturelles des iles Cerbicale et Lavezzi (France, Corse du Sud). Biocosme Mesogeen, 26(1): 1 - 56.

Collins, G. A. 2004. Neuroptera. Br. J. ent. Nat. Hist., 17: 184.

Colombo, R.; Braud, Y.; Danflous, S. 2013. Contribution à la connaissance de Dendroleon pantherinus (Fabricius 1787) (Neuroptera : Myrmeleontidae). Revue de l’Association Roussillonnaise d’Entomologie 22(2): 47-53.

Colombo, R.; Desriau, P.; Gros, P.; Pichard, A. 2012. Redécouverte de deux nouvelles espèces d’ascalaphes (Neuroptera-Ascalaphidae) en France continentale, Deleproctophylla australis (Fabricius, 1787) et Libelloides latinus (Lefèbvre, 1842). Nature de Provence - Revue du CEN PACA, 1 : 123-127.

Contarini. E. 2009. Ulteriori dati sull’entomofauna legata al pino nero (Pinus nigra Arnold) sull’appennino tosco-romagnolo con particolare riguardo alla Val Lamone (Insecta Coleoptera. Neuropteroidea, Hymenoptera). Quaderno di Studi e Notizie di Storia Naturale della Romagna 29:19-36.

Contarini, E.; Mingazzini, A. 2007. Ancora interessanti reperimenti e conferme per l’entomofauna della “Vena del Gesso” romagnola (Insecta Mantodea, Coleoptera, Neuropteroidea, Lepidoptera, Hymenoptera Chrysididae). Quaderno di studi e notizie di Storia Naturale della Romagna, 24: 53-64.

Cooke, D.; Norriss, T.; Fomison, L.; Plant, C. W. 2013. Myrmeleon formicarius (L., 1767) (Neur.: Myrmeleontidae): an ant-lion new to Britain. Entomologist’s Rec. J. Var. 125: 174-178.

Cuppen, J. G. M.; Drost, B. 2005. Entomofauna van Texel verslag van de 159° zomerbijeenkomst te Den Hoorn. Entomologische Berichten 65(3): 70-89.

Cuppen, J. G. M.; Drost, B. 2007. Entomofauna van De Kempen, verslag van de 161° zomerbijeenkomst te Baarschot. Entomologische Berichten 67(4): 122-144.

Cuppen, J. G. M.; Drost, B. 2009. Entomofauna van De noordelijke, verslag van de 163° zomerbijeenkomst te Vorden. Entomologische Berichten 69(5): 169-194.

Cuppen, J. G. M.; Vorst, O. 2004. Entomofauna van Noordoost-Twente verslag van de 158° zomerbijeenkomst te Ootmarsum. Entomologische Berichten 64(6): 188-208.

Czechowska, W. 2000. Sialis morio Klingstedt, 1932 (Megaloptera,Sialidae), an alderfly species new to Poland. Fragmenta Faunistica 43:123-125.

Czechowska, W. 2002. Raphidioptera and Neuroptera (Neuropterida) of the canopy in montane, upland and lowland fir forests of Abies alba Mill. In Poland. Fragmenta Faunistica 45:31-56.

Czechowska, W.; Werstak, K. 2004. Raphidioptera and Neuroptera of the canopy layer in forests of the Swietokrzyski National Park. Fragmenta faunistica 47(2): 97-109.

Deliry, C.; Faton, J.-M. 2010. Histoire naturelle des Ascalaphes de France. Histoires naturelles 10: 3-32.

Dethier, M.; Dopagne, C. 2002. Les insectes aquatiques du ru de Targnon (commune de Theux). Natura Mosana, 54(4):67-76.

Deutsch, B.; Paulian, M.; Thierry, D.; Canard, M. 2005. Quantifying biodiversity in ecosystems with green lacewing assemblages. Agronomy for Sustainable Development 25: 337-343.

Devetak, D. 2002. Neuroptera in oak forests in the submediterranean district of Slovenia. Acta Zoologica Academiae Scientiarum Hungaricae 48 (Suppl. 2), 67-73.

Devetak, D. 2007. A review of the owlflies of Slovenia (Neuroptera: Ascalaphidae). Acta Entomologica Slovenica 15(2): 105-112.

Devetak, D. 2011. Notes on Megaloptera and Neuroptera (Insecta: Neuropterida) of the Brdo Pri Kranju estate (Slovenia). Annales 21: 69-74.

Devetak, D.; Devetak, P. 2004. Neuroleon microstenus (McLachlan, 1898) (Neuroptera: Myrmeleontidae) in the northwestern part of the Balkan Peninsula. Anali za Istrske in Mediteranske Studije [=Annals for Istrian and Mediterranean Studies] 14:55-58.

Devetak, D.; Dobosz, R.; Jaskula, R.; Podlesnik, J.; Klokocovnik, V. 2012. First record of Mantispidae (Neuroptera) from Albania. Acta Entomologica Slovenica 20(2): 153-156.

Devetak, D.; Gomboc, S.; Klokocovnik, V. 2008. Neuropterida of Kozjanski Regional Park, Slovenia. Abstract book, 10th Int. Symp. on Neuropterology, Piran, Slovenia: 31.

Devetak, D.; Jakšič, P. N. 2003. Neuroptera of Kosovo and Metohija (Serbia). Zeitschrift der Arbeitsgemeinschaft Österreichischer Entomologen 55:45-53.

Devetak, D.; Janzekovic, F. 2012. First record of Deleproctophylla australis (Fabricius, 1787)(Insecta: Neuroptera: Ascalaphidae) in Albania. Annales 22: 183-186.

Devetak, D.; Pirš, P.; Janžekovič, F. 2002. Owl-fly Libelloides macaronius (Scopoli, 1763) in Slovenia and in the northwestern part of Croatia (Neuroptera: Ascalaphidae). Annals for Istrian and Mediterranean Studies 12:219-226.

Devetak, D.; Podlesnik, J.; Janžekovič, F. 2010. Antlion Dendroleon pantherinus (Fabricius, 1787) (Neuroptera: Myrmeleontidae) in Slovenia. Acta Entomologica Slovenica 18(2): 159-170.

Devetak, D.; Podlesnik, J.; Klokocovnik, V.; Janzekovic, F. 2013. Antlions (Insecta: Neuroptera: Myrmeleontidae) of Albania. Turkish Journal of Zoology 37: 362-366.

Devetak, P. 2007. New records of two rare neuropterans (Insecta: Neuroptera) in Slovenia. Natura Sloveniae 9(2): 5-10.

Dobosz, R. 2000. Siatkoskrzydłe (Neuropteroidea). Flora i Fauna Pienin – Mon. pienin. 1, 2000: 159-161.

Dobosz, R., 2000. 17. Siatkoskrzydłe (Neuropteroidea) i wojsiłki (Mecoptera) Bieszczadów. Mon. bieszcz. 7, 2000: 245-253.

Dobosz, R., 2001. Trzy nowe dla Puszczy Białowieskiej gatunki owadów siatkoskrzydłych (Neuropteroidea: Megaloptera, Neuroptera). Acta ent. Siles. Vol. VII-VIII, 2001 (1999-2000): 69-70.

Dobosz, R. 2003. Owady siatkoskrzydłe (Neuropterida: Megaloptera, Raphidioptera, Neuroptera) Babiej Góry. [W:] Wołoszyn B.W., Wołoszyn W., Celary W. Monografia Fauny Babiej Góry, 2003: 223-234.

Dobosz, R. 2004. Dendroleon pantherinus (Fabricius, 1787). Polka Czerwona Ksiega Zwierzat – Bezkregowce, 2 pp.

Dobosz, R. 2005. Nowe stanowiska Nothochrysa fulviceps Stephens, 1836 w Polsce (Neuroptera, Chrysopidae). Acta ent. siles., vol. 12-13 (2004-2005): 161.

Dobosz, R. 2010. Sieciarki (Neuroptera). In: Jaskuly, R.; Tonczyka, G. 2010. Owady (Insecta) parku krajobrazowego wzniesień łódzkich. Łódź: 41-43.

Dobosz, R.; Blaik, T. 2008. Lacewings (Neuroptera) of Roztocze – new data and an updated list of species. Acta entomologica silesiana 16:5-11.

Dobosz, R.; Dobosz, M.; Dobosz, M. 2008. Myrmeleon bore Tjeder, 1941 (Neuroptera, Myrmeleontidae) on the Wolin Island with remarks on the biology and laboratory rearing. Acta entomologica silesiana 16: 13-16.

Dobosz, R.; Hadas, T. B. 2009. Entwicklung der neuropterologischen sammlung in lichte der geschichte des oberschlesischen museums in Beuthen. DGaaE Nachr., 23(2):76-78.

Dobosz R., D. Werstak, K.; Czechowska, W. 2005. Raphidioptera of the Pieniny Mountains (S Poland). Ann. Upper Siles. Mus. (Ent.), vol. 13: 71-75.

Dobosz R., D. Żyła. 2005. Drugie stanowisko Myrmeleon inconspicuus Rambur, 1842 w Polsce (Neuroptera, Myrmeleontidae). Acta ent. siles., vol. 12-13 (2004-2005): 161-162.

Dodelin, B. 2005. Compte rendu de la prospection entomologique en Savoie (4 decembre 2004). Bulletin mensuel de la Societe linneenne de Lyon 74(3): 64-66.

Domingo-Quero, T.; Alonso-Zarazaga, M. A.; Sanchez-Ruiz, A.; Araujo Armero, R.; Navas Sanchez, A.; Sanchez Moreno, S.; Garcia Becerra, R.; Nebreda, M.; Sanchez-Ruiz, M.; Fontal-Cazalla, F.; Nieves-Aldrey, J. L. 2003. Inventariando la biodiversidad en el Parque Nacional de la Caldera de Taburiente (La Palma, Islas Canarias, España): novedades cientificas. Graellsia 59(2-3): 45-68.

Dopagne, C.; Dethier, M. 2000. Insects et mollusques aquatiques des etans «Les cours» a Bolland (Pays de Herve, Belgique). Natura Mosana 53:51-64.

Drees, M. 2005. Supplements to the Entomofauna Germanica (Vol. 6)(Insecta: Homoptera, Raphidioptera, Mecoptera). Decheniana 158:101-102.

Dreimanis, J. 2006. Records of ant Lions (Neuroptera: Myrmeleontidae) in Latvia. Latvijas entomologs 43: 129-131.

Drost, B.; Cuppen, J. G. M. 2006. Entomological fauna of the northern part of Drenthe, The Netherlands – report of the 160th summer meeting at Schipborg. Entomol. Ber., 66(3): 70 – 90.

Dubatolov, V.V.; Vasilenko, S.V.; Strelzov, A. N. 2003. New nemoral insect species of Diptera, Coleoptera, Neuroptera, mecoptera, Lepidoptera from the river Argun basin (Chita Oblast’) and their possible zoogeographic significance. Euroasian Entomological Journal 2(3): 167-180.

Duelli, P. 2010. Die ameisenlöwen der Schweiz (Neuroptera, Myrmeleontidae). Entomo Helvetica 3: 155-162.

Duelli, P., Moretti, M.; Tonolla, D.; Barbalat, S. 2006. Scented traps yield two large lacewing species (Neuroptera, Chrysopidae) new to Switzerland. Mitt. Schweiz. Entomol. Gesell., 79:25-28.

Dunk, von der K. 2006. Kommentierte Insektenliste der Abteilung Schiessturmlach im Heroldsberger Forst / N-Bayern. Galathea, 22(3):85-114.

Dunk, von der K. 2006. Vorkommen bemerkenswerter Insecktenarten im bereich des Markwaldes, nördlich von Erlangen, Mittelfranken. Galathea, 22(4):157-173.

Dunk, K., von der; Amon, F. J. 2002. Untersuchungen zur Insektenfauna auf der Stromleitung-Sandtrasse Schwaig bei Nürnberg (Kreis Nürnberger Entomologen e. V.). Galathea 18:145-163.

Dunk, K., von der; Brünner, K. 2013. Neue erkenntnisse über futterkugel von mauerseglern aus Ostmittelfranken / Bayern / BRD (Aves: Apus apus L. und Insecta varia). Galathea 29:119-145.

Dunk, K., von der; Köstler, W. 2002. Biocenosis of wood inhabiting insects in the honeysuckle Lonicera xylosteum L. Galathea Supplement 11:89-92.

Dunk, K., von der; Köstler, W.; Tannert, R.; Weltner, L. 2011. Erfassung der insektenfauna der Nürnberger Kaiserburg für das Projekt „Lebensraum Burg“ im rahmen der Umsetzung der Bayerischen biodiversitätsstrategie. Galathea 27(3): 93-140.

Dunk, K., von der; Köstler, W.; Tannert, R.; Weltner, L. 2012. Fortsetzung der erfassung der insektenfauna für da projekt „Lebensraum Burg“ rings un die Nürnberger Kaiserburg. Galathea 28: 53-79.

Dunk, K., von der; Niedling, A. 2003. Einblicke in die insektenfauna einer Sandackerbrache. Galathea 19(4): 169-188.

Dunk, K., von der; Weltner, L. 2013. Untersuchung der beifänge im areal der Kaiserburg Nürnberg. Galathea 29:75-99.

Endrestøl, A. 2011. Survey of the antlion Myrmeleon bore 2011 in Norway. NINA Report 765, 68 pp.

Fernández-Cardenete, J. R.; Tierno De Figueroa, J. M. 2002. Primera cita de Osmylus fulvicephalus (Scopoli, 1763) en el sur de la Peninsula Iberica (Planipennia, Osmylidae). Boletin de l’Asociacion espanola de Entomologia 26 (1-2):199-200.

Fesl, C.; Humpesch, U. H.; Wöss E. R. 2005. Biodiversity of the macrozoobenthos of the Austrian Danube, including quantitative studies in a free-flowing stretch below Vienna. Denisia 16: 139-158.

Fischer, U.; Weigel, A. 2000. Beitrag zur Fauna des Geschützten Landschaftsbestandteiles “Weise am Kirchsteig” und dessen Umfeld bei Niederböhmersdorf (Thüringen: Landkreis Greiz). Thüringer Faunistische Abhandlungen 7:21-44.

Fogh Nielsen, O. 2004. Peyerimhoffina gracilis (Schneider, 1851) recorded as new to Denmark (Neuroptera, Chrysopidae). Entomologiske Meddelelser 72:31-32.

Fogh Nielsen, O. 2013. The lacewing Peyerimhoffina gracilis (Schneid., 1851) now established in Denmark (Neuroptera, Chrysopidae). Entomologiske Meddelelser 81(2):85-87.

Franchi, M.; De Maglio, G.; Miani, N. 2008. Studio sulla qualità biologica delle acque del fiume Natisone e dei suoi principali affluenti (Italia nord-orientale). Gortiana, Atti del Museo Friulano di Storia Naturale 29: 203-256.

Frankenhuyzen, A. van; Stigter, H. 2002. Schädliche und nützliche insekten und Milben an Kern- und Steinobst. Ulmer Verlag, Stuttgart, 288 pp.

Frapa, P. 2011. 24 heurs naturalistes d’Entrevennes (4-6 juin 2010) (Alpes-de-Haute-Provence - France). Synthèses des connaissances faunistiques et floristiques. Entrevennes Anim’action, Entrevennes 122 pp.

Frapa, P.; Lamoline, J.-P.; Braud, Y. 2012. Synthèse des connaissances faunistiques sur la commune d’Entrevennes (Alpes-de-Haute-Provence - France) corrigenda & addenda. Entrevennes, 16 pp.

Garcia, A.; Figueiredo. E.; Valente, C.; Monserrat, V.J.; Branco, M. 2013. First record of Thaumastocoris peregrinus in Portugal and of the neotropical predator Hemerobius bolivari in Europe. Bulletin of Insectology 66(2): 251-256.

Gavira, O.; Sanchez, S.; Carrasco, P.; Ripoll, J.; Solis, S. 2012. Presence of the family Nevrorthidae (Neuroptera) in the Iberian peninsula. Boletin de la Sociedad Entomologica Aragonesa 51: 217-220.

Giacomino, M. 2002. Notes de chasses et observations diverses – Neuroptera: complement de localisation. Entomologiste 58(5-6): 283-284.

Giacomino, M. 2004. Complement de localisation sur quelques Nevropteres (Mantispidae et Chrysopidae) en France metropolitaine. Entomologiste 60(6): 249-250.

Giacomino, M. 2010. Aleuropteryx iberica Monserrat, 1977. Coniopterygidae nouveau pour la Faune de France (Insecta, Neuroptera). Revue de l’Association Roussillonnaise d’Entomologie 19(1): 28-29.

Giacomino, M. 2010. Mise a jour de la faune des Myrmeleontinae (Neuroptera, Myrmeleontidae) des Pays de la Loire (France). Bull. Soc. Sc. Nat. Ouest de la France, n.s. 32(4): 209-215.

Giacomino, M. 2011. Wesmaelius (Kimminsia) tjederi (Kimmins, 1963). Hemerobiinae nouveau pour la Faune de France (Neuroptera, Hemerobiidae). Revue de l’Association Roussillonnaise d’Entomologie 20(1): 13-16.

Giacomino, M. 2012. Wesmaelius (Kimminsia) navasi (Andréu, 1911). Hemerobiinae nouveau pour la Faune de France (Neuroptera, Hemerobiidae). Revue de l’Association Roussillonnaise d’Entomologie 21(2): 60-61.

Giacomino, M. 2013. Contribution à la connaissance des Neuropterida de Corse (Raphidioptera et Neuroptera). Micromus angulatus (Stephens, 1836), Microminae nouveau pour la faune de Corse (Neuroptera Hemerobiidae). L’Entomologiste 69(3): 117-120.

Głowaciński, Z.; Nowacki, J. (eds) 2004. Polish Red Data Book of Animals. Invertebrates. Instytut Ochrony Przyrody PAN, Krakowie, 448 pp. (cfr. pp. 81-88).

Gossner, M. 2002. Vergleichende waldökologische Untersuchungen in Naturwaldreservaten (ungenutzten Wäldern) und Wirtschaftswäldern unterschiedlicher Naturnähe (unter Einbeziehung der Douglasie) in Mittelschwaben. Teil 4 Arthropoden auf Neophyten. Forschungsvorhaben des BMBF (0339735A) und der bayerischen Staatsforstverwaltung (L51), 216 pp.

GRETIA. 2009. Etat des lieux des connaissances sur les invertébrés continentau des Pays de la Loire ; bilan final. Rapport GRETIA puor le Conseil Régional des Pays de la Loire. 396 pp.

Greve, L. 2002. The order Raphidioptera in Norway. Norwegian Journal of Entomology 49:81-92.

Greve, L. 2004. Micromus variegatus (Fabricius 1793) (Neuroptera, Hemerobiidae) in Norway. In: Aspöck, U. (eds.): Entomologie und Parasitologie - Festschrift zum 65. Geburtstag von Horst Aspöck, 640 pp. Denisia 13: 183-184.

Greve, L. 2006. Nebbfluer, kamehalsfluer, mudderfluer og nettvinger. 231-235. In: Kalas, J. A.; Viken, A.; Bakken, T. (eds). Norsk Rodliste 2006. Artsdatabanken, Norway, 416 pp.

Greve, L. 2007. Nebbfluer, kamehalsfluer, mudderfluer og nettvinger. Insekt-Nytt 32(1/2): 38-41.

Greve, L.; Andersen, T. 2012. Lacewing (Neuroptera) and alderflies (Megaloptera) from Finnmark, northern Norway. Norwegian Journal of Entomology 59:122-132.

Greve, L.; Kobro, S. 2009. Abundance of Micromus variegatus (Schneider, 1851) (Neuroptera, Hemerobiidae) in SE Norway as indicated by light-trap catches. Norwegian Journal of Entomology 56:140-142.

Greve, L.; Olsen, K. M.; Kobro, S. 2011. Peyerimhoffina gracilis (Fabricius 1793) (Neuroptera, Chrysopidae) new to Norway. Norwegian Journal of Entomology 58(2):126-127.

Grilli, P. ; Letardi, A. ; Balsamo, M. 2009. Rinvenimento nelle Marche di Sialis fuliginosa Pictet, 1836. Quaderno di Studi e Notizie di Storia Naturale della Romagna 28:141-148.

Gruppe, A. 2002. Verbreitung der Taxa des Chrysoperla carnea- Komplex in Südbayern (Neuroptera: Chrysopidae). Galathea 13 suppl.: 15-19.

Gruppe, A. 2005. Neuropteren in Baumkronen – Methodik und Ergebnisse. Galathea 18 suppl.: 7-12.

Gruppe, A. 2005. Neue Nachweise von Nineta principiae MONTSERRAT 1980 in Bayern (Neuroptera, Chrysopidae). Nachrichten der Deutsche Gesellschaft für allgemeine und angewandte Entomologie e.V. 19(3):139-140.

Gruppe, A. 2007. Neuropteren im Englischen Garten, München. Nachrichten der Deutsche Gesellschaft für allgemeine und angewandte Entomologie e.V. 21(3): 138-139.

Gruppe, A. 2007. Ungewöhnliche Funde von Neuropteren. Nachrichten der Deutsche Gesellschaft für allgemeine und angewandte Entomologie e.V. 21(3): 141-142.

Gruppe, A. 2013. Neuropteren auf nicht einheimischen Baumarten. Nachrichten der Deutsche Gesellschaft für allgemeine und angewandte Entomologie e.V. 27: 21-23.

Gruppe, A.; Gossner, M.; Simon, U. 2004. Neuropteren in Wäldern Schwabens und Oberbayerns (Insecta: Neuropterida). Beiträge zur bayerischen Entomofaunistik 6: 243-254.

Gruppe, A.; Gossner, M.; Späth, J. 2008. Neunachweise von Psectra diptera (Burmeister, 1839) in Bayern (Neuroptera: Hemerobiidae). NachrBl. Bayer. Entomol. 57(1/2): 26-29.

Gruppe, A.; Müller, J. 2007. Distribution of Neuropterida in beech dominated forests in southern Germany. Ann. Mus. civ. St. nat. Ferrara, 8: 145-152.

Gruppe, A.; Zehetmair, T. 2009. Nachweise von Coniopterygiden in Bayern. Nachrichten der Deutsche Gesellschaft für allgemeine und angewandte Entomologie e.V., 23(2):85-86.

Hable, J.; Dunk, K. v.D.; Knipfer, G. 2010. Zur entomofauna primärer felsheiden und blockschutthalden im südlichen landkreis Neumarkt/Opf. Galathea suppl. 19: 1-104.

Harvey, M. C. 2004. Sisyra terminalis Curtis (Neuroptera: Sisyridae) at Richmond Park, Surrey, in 1994. British Journal of Entomology and Natural History 17: 44.

Háva, J. 2000. The genus Deleproctophylla Lefebvre, 1842 (Insecta: Neuroptera: Planipennia: Ascalaphidae) from the collection of the department of Entomology, National Museum Prague. Casopis Narodniho muzea Rada prirodovedna 169:16.

Háva, J. 2000. The genus Libelloides Tjeder (Insecta: Neuroptera: Planipennia: Ascalaphidae) from the collection of the department of Entomology, National Museum Prague. Casopis Narodniho muzea Rada prirodovedna 169:101-104.

Heidger, C. M. 2007. Die Ökologie von Ameisenlöwen (Myrmeleon bore, Euroleon nostras) auf Sukzessionsflächen in der Oberlausitz. Nachrichten der Deutsche Gesellschaft für allgemeine und angewandte Entomologie e.V. 21(3): 136-137.

Hellrigl, K. 2006. Zur vorkommen der heimischen Netzflügler (Neuropteroidea). Forest Observer 2/3 (2006): 490-494.

Henry C.S., Brooks S.J., Duelli P., Johnson J.B., 2002. Discovering the true Chrysoperla carnea (Insecta: Neuroptera: Chrysopidae) using song analysis, morphology, and ecology. Ann. Entomol. Soc. Am. 95(2):172-191.

Henry C.S., Brooks S.J., Duelli P., Johnson J.B., 2003. A lacewing with the wanderlust: the European song species 'Maltese', Chrysoperla agilis sp.n., of the carnea group of Chrysoperla (Neuroptera: Chrysopidae). Syst. Entomol., 28: 131 - 147.

Hoffrichter, O.; Tröger, E. J. 2006. Weitere insekten am Schönberg. In: Körner, H. 2006. Der Schönberg. Natur- und Kulturgeschichte eines Schwarzwald-Vorberges. Lavori Verlag, Freiburg im Breisgau, 421 pp.

Holuša, J.; Vidlička, L. 2002. Chrysopids and Hemerobiids (Planipennia) of young spruce forests in eastern part of the Czech Republic. Journal of Forest Science 48(10): 432-440.

Hölzel, H. 2002. Insecta: Megaloptera. Süsswasserfauna von Mitteleuropa 15.

Hölzel H., Monserrat V.J., 2002 – Tricholeon relictus nov. sp. – a new European antlion (Neuropterida: Neuroptera: Myrmeleontidae). Linzer biol. Beitr., 34(2): 1151 - 1154.

Hölzel, H.; Weissmair, W. 2002. Insecta: Neuroptera. Süsswasserfauna von Mitteleuropa 16.

Hondt, J.-L. d’. 2006. Notes sur les Ascalaphes de Dordogne (Neuroptera Ascalaphidae). Entomologiste 62(3-4): 93-95.

Hondt, J.-L. d’. 2007. Neuropteres en Perigord. Entomologiste 63(1): 23-25.

Ilbert, N.; Menegaux, J. 2007. Observations d’insectes dans le Hautes-Pyrenees et les Pyrenees-Orientales (Orthoptera, Odonata, Lepidoptera et Neuroptera). Entomologiste 63(1): 46.

Insom, E.; Terzani, F. 2012. Nuovi dati di Rafidiotteri e Neurotteri per l’Arcipelago Toscano (Insecta Raphidioptera Raphidiidae, Neuroptera Chrysopidae, Myrmeleontidae). Quaderno di Studi e Notizie di Storia Naturale della Romagna 35: 157-161.

Jacquemin, G. 2007. Listes de référence des insectes de Lorraine. 2. Neuropteroidea Mecoptera. Bulletin et Listes de Référence de la Société lorraine d'Entomologie: 26pp.

Jacquemin, G. 2013. Sisyra dalii McLachlan, 1866, la troisième espèce de Sisyra retrouvée (Neuroptera, Sisyridae). Bulletin de la Société lorraine d'Entomologie 14: 23-24.

Jacquemin, G.; Claude, A. 2012. Inocellia crassicornis (Schummel, 1832) en Lorraine, nouvelle espèce pour la France (Raphidioptera Inocelliidae). Entomologiste 68(2): 73-75.

Jacquemin, G.; Dabry, J. 2013. Micromus (Nesomicromus) lanosus (Zelený, 1962), espèce nouvelle pour la Lorraine, et intéressante donnée française (Neuroptera, Hemerobiidae). Bulletin de la Société lorraine d'Entomologie 14: 30-31.

Jedlicka, L.; Sevcik, J.; Vidlicka, L. 2004. Checlist of Neuroptera of Slovakia and Czech Republic. Biologia, Bratislava, 59(suppl.15):59-67.

Jones, J.R.; Devetak, D. 2009. First record of Nevrorthidae from Slovenia. Acta Entomologica Slovenica 17(2): 99-106.

Jones, J. R.; Magalhães, B.I.; Saraiva, J.; Gruppe, A. 2013. Post-symposium excursion to natural areas of mainland Portugal. In: Martins, A.M.F., & M.A.M. Ventura (Eds.), Proceedings of the Eleventh International Symposium of Neuropterology, Ponta Delgada, São Miguel, Açores, June 13-15, 2011. Açoreana, Suplemento 9: 143-155.

Jordal, J. B.; Grimstad, K. J.; Holtan, D. 2003. Maurløva Myrmeleon formicarius i Møre og Romsdal. Insekt-Nytt 19(3):15-17.

Kacirek, A. 2013. Contribution to the faunistics of antlions (Neuroptera: Myrmeleontidae) of Bulgaria, Macedonia and Greece. Klapalekiana 49:189-196.

Kalniņš, M. 2002. Raphidioptera. www.lubi.edu.lv/les/Raphidioptera.htm .

Kalniņš, M. 2002. Megaloptera. www.lubi.edu.lv/les/Megaloptera.htm .

Kalniņš, M. 2002. Neuroptera. www.lubi.edu.lv/les/Neuroptera.htm .

Kemp, R. J. 2000. Neuroptera. British Journal of Entomology and Natural History 13:186.

Klausnitzer, B. 2005. Die Insektenfauna Deutschlands („Entomofauna Germanica“) – ein Gesamtüberblick. Linzer biol. Beitr., 37(1): 87-97.

Klokocovnik, V.; Devetak, D.; Gomboc, S. L. 2010. Neuropterida (Megaloptera, Raphidioptera, Neuroptera) of Kozjanski Regional Park, Slovenia. In: Devetak, D.; Lipovsek, S.; Arnett, A.E. (eds) 2010. Proceedings of the 10th Int. Symp. on Neuropterology. Maribor, Slovenia: 171-174.

Kovanci, B.; Kovanci, O. B. 2007. An annotated list of the green lacewings (Neuroptera: Chrysopidae) of Northwestern Turkey, with new records, their spatio-temporal distribution, and harbouring plants. Entomological News 118(1): 90-104.

Krivokhatsky, V.A. 2002. Neuropterida. pp. 27, 162-169. In: Noskov (eds). Red data book of nature of the Leningrad region. St. Petersburg.

Kumanski, K.; Popov, A. 2000. Megaloptera and Trichoptera (Insecta) of glacial waters in the Rila Mountains, Bulgaria. In: Golemansky V., W. Naidenow (eds). Biodiversity and evolution of glacial water ecosystems in the Rila Mountains. Sofia, Ins. Zool., 61-66.

Lazzara, M.; Lencioni, V.; Maiolini, B. 2006. Macroinvertebrati. 185-207. In: Cantonati, M.; Lazzara, M. (eds). I laghi di alta montagna del bacino del fiume Avisio (Trentino orientale). Monografie del Museo Tridentino di Scienze Naturali 3: 249 pp.

Lequet, A. ; Faucheux, M. J. 2006. La Mantispe de Styrie, Mantispa styriaca Poda (Neuroptera : Mantispidae) en Vendée (85), France : attitudes comportamentales. Bull. Soc. Sc. Nat. Ouest de la France, N.S. 28(4) : 213-219.

Letardi, A. 2000. Reperti. Neuroptera, Sisyridae, Hemerobiidae, Myrmeleontidae, Ascalaphidae. Bollettino dell’Associazione Romana di Entomologia, 55(2000): 134-145.

Letardi, A. 2002. Megaloptera, Neuroptera, Mecoptera p.105. In: MASON F., CERRETTI P., TAGLIAPIETRA A., SPEIGHT M.C.D., ZAPPAROLI M. (eds). Invertebrati di una foresta della Pianura Padana, Bosco della Fontana, Primo contributo. Conservazione Habitat Invertebrati 1. Gianluigi Arcari Editore, Mantova, 176 pp.

Letardi, A. 2003. Neuropterida, Mecoptera p.122-123. In: CERRETTI P., TAGLIAPIETRA A., TISATO M., VANIN S., MASON F., ZAPPAROLI M. (eds). Artropodi dell’orizzonte del faggio nell’Appennino Settentrionale, Primo contributo. Conservazione Habitat Invertebrati 2. Gianluigi Arcari Editore, Mantova, 256 pp.

Letardi, A. 2004. I formicaleoni (Insecta Neuroptera Myrmeleontidae) delle aree costiere italiane. Atti dei Convegni Lincei 205: 293-298.

Letardi, A. 2004. Turcoraphidia amara (Aspöck et Aspöck, 1964) genere e specie nuovi per l’Italia (Raphidioptera). Bollettino dell’Associazione romana di Entomologia 59: 99-102.

Letardi A., 2004. Short notes 56. Megaloptera. p.298. In: Cerretti, P.; Hardersen, S.; Mason, F.; Nardi, G.; Tisato, M.; Zapparoli, M. (eds). 2004. Invertebrati di una foresta della Pianura Padana, Bosco della Fontana. Secondo contributo. Conservazione Habitat Invertebrati, 3. Cierre Grafica Editore, Verona, 304 pp.

Letardi A., 2004. Short notes 57. Neuroptera. p.298-299. In: Cerretti, P.; Hardersen, S.; Mason, F.; Nardi, G.; Tisato, M.; Zapparoli, M. (eds). 2004. Invertebrati di una foresta della Pianura Padana, Bosco della Fontana. Secondo contributo. Conservazione Habitat Invertebrati, 3. Cierre Grafica Editore, Verona, 304 pp.

Letardi, A. 2005. Insecta Neuroptera, Megaloptera e Raphidioptera. In: Ruffo, S.; Stoch, F. (eds) 2005. Checklist e distribuzione della fauna italiana. Memorie del Museo Civico di Storia Naturale di Verona, 2 serie, Sezione Scienze della Vita 16: 235-236.

Letardi, A. 2007. Rafidiotteri, Neurotteri e Mecotteri del Parco e delle aree adiacenti (Raphidioptera, Neuroptera, Mecoptera), pp. 297-305. In: Nardi G. & Vomero V. (eds.), Artropodi del Parco Nazionale del Vesuvio: ricerche preliminari. Conservazione Habitat Invertebrati, 4. Cierre edizioni, Verona.

Letardi, A. 2007. Reperti. Neuroptera, Coniopterygidae, Hemerobiidae. Bollettino dell’Associazione Romana di Entomologia 62(1-4): 145-146.

Letardi, A. 2009. Neurotteridi e Mecotteri delle Riserve Naturali "Agoraie di Sopra e Moggetto" (Liguria, Genova) e "Guadine Pradaccio" (Emilia-Romagna, Parma) (Megaloptera, Raphidioptera, Neuroptera, Mecoptera). Bollettino dell’Associazione Romana di Entomologia 64(1-4): 281-291.

Letardi, A.; Almeida, J. M.; Badano, D.; Silva, R. R.; Machado, E. 2013. Contributing to a checklist of Neuropterida in Portugal: the Naturdata project. In: Martins, A.M.F., & M.A.M. Ventura (Eds.), Proceedings of the Eleventh International Symposium of Neuropterology, Ponta Delgada, São Miguel, Açores, June 13-15, 2011. Açoreana, Suplemento 9: 29-38.

Letardi, A.; Aspöck, U.; Aspöck, H.; Pantaleoni, R.A. 2006. Nevrorthus apatelios H.Aspöck et U.Aspöck et Hölzel, 1977 (Neuroptera Nevrorthidae) nelle Prealpi Friulane. Rivista del Museo civico di Scienze Naturali “E. Caffi” di Bergamo 24: 91-92.

Letardi, A.; Bartolozzi, L. 2007. Segnalazioni di Sympherobius (Niremberge) klapaleki Zeleny, 1963 (Neuroptera Hemerobiidae). Onychium 5: 45-47.

Letardi, A.; Biscaccianti, A. B. 2007. Neuropterida of Majella National Park, Italy. Annali del Museo civico di Storia naturale, Ferrara 8: 107-110.

Letardi, A.; Cristofaro, M. 2005. Nota su alcuni Neuroptera di Toscana, Umbria e Marche (Insecta Neuropterida)(Italia). Proceedings XX Congresso Nazionale Italiano di Entomologia, Perugia-Assisi 13-18 Giugno 2005 453 pp: 87.

Letardi, A.; Maltzeff, P. 2001. Neurotteridi e Mecotteri della Tenuta Presidenziale di Castelporziano e delle aree limitrofe (Neuroptera, Raphidioptera, Mecoptera). Bollettino dell'Associazione Romana di Entomologia 54:49-62.

Letardi, A.; Maltzeff, P. 2008. I Neuropterida della Tenuta Presidenziale di Castelporziano. Addenda (Neuroptera, Raphidioptera). Bollettino dell'Associazione Romana di Entomologia 63:83-89.

Letardi, A & E. Migliaccio 2002. Neuropterida of the Abruzzo National Park, Italy. Acta Zoologica Academiae Scientiarum Hungaricae 48 (Suppl. 2), 149-154.

Letardi, A.; Nicoli Aldini, R. 2007. Contributo alla conoscenza dei Neuropterida del Molise (Raphidioptera, Megaloptera, Neuroptera). Bollettino dell’Associazione Romana di Entomologia 62(1-4): 111-130.

Letardi, A.; Nicoli Aldini, R.; Pantaleoni, R. A. 2010. The Neuropterida of Triveneto (Northern Italy): an updated faunal checklist with some zoogeographical remarks. In: Devetak, D.; Lipovsek, S.; Arnett, A.E. (eds) 2010. Proceedings of the 10th Int. Symp. on Neuropterology. Maribor, Slovenia: 181-189.

Letardi, A.; Pace, G. 2000. Reperti. Megaloptera, Sialidae. Bollettino dell’Associazione Romana di Entomologia, 55(2000): 144.

Letardi, A.; Teixeira, A.; Oliveira, J. 2013. A faunal and historical survey of Sialidae (Insecta: Megaloptera) in continental Portugal. In: Martins, A.M.F., & M.A.M. Ventura (Eds.), Proceedings of the Eleventh International Symposium of Neuropterology, Ponta Delgada, São Miguel, Açores, June 13-15, 2011. Açoreana, Suplemento 9: 21-27.

Letardi, A.; Thierry, D.; Tillier, P.; Canard, M. 2008. Mise à jour de la faune des neuropterida de Corse (Raphidioptera & Neuroptera). Revue de l’Association Roussillonnaise d’Entomologie 17(3): 95-105.

Lipovšek, S & B. Mencinger 2002. Remarks on Neuroptera of north-eastern Slovenia (Goričko). Acta Zoologica Academiae Scientiarum Hungaricae 48 (Suppl. 2), 159-163.

Lock, K.; San Martin, G. 2013. Checklist of the Belgian Neuroptera. Bulletin de la Société royale belge d’Entomologie 149: 233-239.

Longo, S.; Barbagallo, S.; Rapisarda, C.; Tropea Garzia, G.; Mazzeo, G.; Siscaro, G.; Bella, S. (with contributions of Russo, A.; Suma, C.; Nucifora, S.; Pulvirenti, A.; Inserra, S.; Pantaleoni, R.; Letardi, A.; Patti, I.; Cocuzza, G.) 2001. Note sull’artropodofauna degli ambienti forestali e agrari del Parco dell’Etna. Tecnica Agricola, 53 (3-4):3-61.

Loru, L.; Letardi, A.; Pantaleoni, R.A. 2011. Neuropterida dell'Iglesiente (Sardegna sudoccidentale) (Raphidioptera, Neuroptera). In: Nardi G., Whitmore D., Bardiani M., Birtele D., Mason F., Spada l. & Cerretti P. (eds), Biodiversity of Marganai and Montimannu (Sardinia). Research in the framework of the ICP Forests network. Conservazione Habitat Invertebrati 5: 759-766.

Makarkin, V. N.; Klepikov, M. A. 2012. New records of Neuroptera and Raphidioptera from Yaroslavskaya and Kostromskaya Oblasts. Euroasian Entomological Journal 12(6): 570-574.

Makarkin, V. N.; Tshistjakov, Yu. A. 2009. The Dilaridae (Neuroptera): poorly known “pleasing” lacewings. Eversmannia 19-20: 36-47.

Marcuzzi, G. 2003. Fauna della Provincia di Belluno. Studi Trentini di Scienze Naturali - Acta Biologica 79:121-172.

Mazel, R. 2001. Notes sur les Ascalaphidae du sud de la France (Neuroptera). R.A.R.E., 10(1):3-7.

Mazel, R. 2003. Observation d'Italochrysa stigmatica (Rambur) en France et de quelques autres Chrysopidae (Nevroptera, Notochrysinae - Chrysopinae). R.A.R.E., 12(1):35-37.

Mazel, R.; Canard, M.; Thierry, D. 2006. Cles synoptiques des Chrysopidae de France (Neuroptera). Revue de l’Association Roussillonnaise d’Entomologie 15(1): 29-45.

Mignon, J. 2002. Quelques Nevropteres d’un site de la region liegeoise (ru de Targnon, Theux, Belgique). Notes fauniques de Gembloux 47:69-70.

Miguélez, A.; Valladares, L. F. 2008. Contribucion al conocimiento de Osmylus fulvicephalus (Scopoli, 1763) en el area cantabrica: distribucion y caracteristicas del habitat de su larva (Planipennia, Osmylidae). Graellsia 64(2): 345-348.

Molinu, A.; Sassu, A.; Pantaleoni, R. A. 2007. Neuropterida of the Asinara Island (NW Sardinia, Italy). Annali del Museo civico di Storia naturale, Ferrara 8: 111-115.

Monserrat, V. J. 2002. New data on the dusty wings from Africa and Europa (Insecta, Neuroptera, Coniopterygidae). Graellsia 58:3-19.

Monserrat, V. J. 2004. Nuevos datos sobre algunas especies de hemeróbidos (Insecta: Neuroptera: Hemerobiidae). Heteropterus : Revista de Entomología 4:1-26.

Monserrat, V. J. 2005. Catalogo de los Neuropteros de Baleares con nuevos datos sobre su fauna (Insecta, Neuroptera). Boll. Soc. Hist. Nat. Balears, 48: 71-85.

Monserrat, V. J. 2005. Nuevos datos sobre algunas pequeñas familias de neuropteros (Insecta: Neuroptera: Nevrorthidae, Osmylidae, Sisyridae, Dilaridae). Heteropterus : Revista de Entomología 5:1-26.

Monserrat, V. J. 2006. Nuevos datos sobre algunas especie de la familia Berothidae (Insecta: Neuroptera). Heteropterus : Revista de Entomología 6:173-207.

Monserrat, V. J. 2008. Nuevos datos sobre algunas especies de Nemopteridae y Crocidae (Insecta: Neuroptera). Heteropterus : Revista de Entomología 8(1):1-33.

Monserrat, V. J. 2008. Nuevos datos sobre algunas especies de crisopidos (Insecta: Neuroptera: Chrysopidae). Heteropterus : Revista de Entomología 8(2):171-196.

Monserrat, V. J. 2008. Nuevos datos sobre algunas especies de Hemerobidos (Insecta, Neuroptera, Hemerobiidae). Graellsia 62(2):233-253.

Monserrat, V. J. 2010. Nuevas o interesantes citas de neuropteros en la Peninsula Iberica (Insecta: Neuroptera). Heteropterus Revista de Entomologia 10(1): 19-34.

Monserrat, V. J. 2011. Sobre algunas especies de neurópteros de la península Ibérica y de las Islas Canarias de posición taxonómica problemática o con citas dudosas o cuestionables (Insecta, Neuroptera: Megaloptera, Planipennia). Boletín de la Sociedad Entomológica Aragonesa 49: 153-178.

Monserrat, V. J. 2013. Los Neurópteros (Neuroptera). 283-309 + 515-516. In: Ruano, F.; Tierno de Figueroa, M.; Tinaut, A. 2013. Los Insectos de Sierra Nevada. 200 años de historia. Asociación Española de Entomología. Vol. 1: 544 pp.

Monserrat, V. J.; Acevedo, F. 2011. Nuevos datos sobre las hormigas léon de la Peninsula Ibérica (Insecta: Neuroptera: Myrmeleontidae). Heteropterus Revista de Entomologia 11(1): 123-136.

Monserrat, V. J.; Acevedo, F. 2013. Los mirmelonidos (hormigas-léon) de la Peninsula Ibérica e islas Baleares (Insecta, Neuropterida, Neuroptera, Myrmeleontidae). Graellsia 69(2): 283-321.

Monserrat, V. J.; Acevedo, F.; Triviño, V. 2012. Los ascalafidos de la Peninsula Iberica y Baleares (Insecta: Neuroptera: Ascalaphidae). Heteropterus Revista de Entomologia 12(1): 33-58.

Monserrat, V. J.; Papenberg, D. 2006. Revision del genero Harraphidia Steinmann, 1963 con la descripcion de dos nuevas especies de la Peninsula Iberica y de Marruecos (Insecta, Raphidioptera). Graellsia 62(2):203-211.

Monserrat, V. J.; Papenberg, D. 2010. Revision del genero Phaeostigma Navàs, 1909 de la Peninsula Iberica (Insecta, Raphidioptera). Graellsia 66(1):47-77.

Monserrat, V. J.; Papenberg, D. 2012. Revision del genero Venustoraphidia Aspöck & Aspöck, 1968 de la Peninsula Iberica (Insecta, Raphidioptera). Graellsia 68(2):291-304.

Monserrat, V. J.; Triviño, V. 2013. Atlas de los neuropteros de la Peninsula Iberica e Islas Baleares (Insecta, Neuroptera: Megaloptera, Raphidioptera, Planipennia). Monografias de la Sociedad Entomologica Aragonesa 13: 1-154.

Monserrat, V. J.; Triviño, V.; Acevedo, F. 2012. Los nemopteridos y crocidos de la Peninsula Iberica y Baleares (Insecta: Neuroptera: Nemopteridae, Crocidae). Heteropterus Revista de Entomologia 12(2): 231-255.

Monserrat, V. J.; Triviño, V.; Acevedo, F. 2013. Contribución al conocimiento de los neurópteros de Navarra (Insecta: Neuroptera). Heteropterus Revista de Entomologia 13(1): 41-58.

Monserrat, V. J.; Triviño, V.; Acevedo, F.; Garcia, A. 2013. Nuevos datos sobre algunas especies de Hemerobidos de la peninsula Iberica e Islas Canarias, incluyendo una nueva especie invasora de origen neotropical en Portugal (Insecta, Neuroptera, Hemerobiidae). Graellsia 69(2): 157-168.

Morin, D. 2001. Capture d’Acanthaclisis occitanica (Villiers, 1789) à Montpellier (Neuroptera, Myrmeleontidae, Myrmeleontinae). R.A.R.E., 10(1):2.

Morin, D. ; Maldes, J.-M. 2001. Les lapiaz d’Argelliers : bonne localité pour les Ascalaphidae (Neuroptera). R.A.R.E., 10(1):1.

Mukkala, V.-M.; Haarto, A.; Koponen, S.; Mukkala, L.; Rinne, V.; Salmela, J. 2005. On insects, arachnids and other invertebrates in Kivistönmäki, Ilmajoki. W-album 2: 3-34.

Müller, M.; Krüsi, B.O.; Schlegel, J. 2013. Raumnutzung des libellen-schmetterlingshafts nördlich der Alpen. Folgerungen für den Artenschutz. Naturschutz und Landschaftsplanung 45(1): 21-28.

Müller, M.; Schlegel, J.; Krüsi, B.O. 2012. The owly sulphur Libelloides coccajus (Denis & Schiffermüller, 1775)(Neuropterida: Neuroptera: Ascalaphidae) in the canton of Aargau: actual distribution and recommendations for species conservation. Mitteilungen der Schweiserischen Entomologischen Gesellschaft 85: 177-199.

Müller, R.; Weissmair, W. 2012. Rediscovery of Sisyra jutlandica in Germany and first record of Sisyra dalii for Brandenburg (Insecta, Neuroptera, Sisyridae). Lauterbornia 74: 63-72.

Nash, D. R. 2009. The giant lacewing Osmylus fulvicephalus Scopoli (Neuroptera) new to Suffolk – a very belated record. Suffolk Natural History 45: 33-34.

Nicoli Aldini, R. 2005. I Neurotteri (Insecta Neuropterida) della Val Canonica e del Parco Naturale dell’Adamello (alpi e Prealpi Lombarde): materiali per un’analisi zoogeografica del popolamento. Biogeographia 26: 417-430.

Nicoli Aldini, R. 2007. Presenza di Psectra diptera (Burmeister, 1839)(Neuroptera Hemerobiidae) nell’Italia appenninica. Proceedings XXI Congr. Naz. It. Entomol., Campobasso 11-16 giugno 2007: 63.

Nicoli Aldini, R. 2007. Insetti e altri invertebrati, pp. 56-76. In: Momeliano e il Rio Gandore di Gazzola, un itinerario nella biodiversità (a cura di C. Lorenzoni e P. Cravedi), Comune di Gazzola e Università Cattolica del Sacro Cuore, Officina Foto Grafica, Piacenza, 80 pp. (Neurotteri: pp. 63-64).

Nicoli Aldini, R. 2012. Lacewings (Neuroptera) as beneficial insects in orchards: findings for plum and cherry trees in Lombardy (northern Italy). IOBC/wprs Bullettin 74: 203-208.

Nicoli Aldini, R.; Baviera, C. 2001. Nuove acquisizioni sulla Neurotterofauna di Sicilia e isole circumsiciliane (Insecta Neuropterida). Naturalista siciliano, S. IV, 25(3-4):345-376.

Nicoli Aldini, R.; Letardi, A.; Pantaleoni, R. A. 2012. State of the art on Neuropterida of Sicily and Malta. Biodiversity Journal 3(4): 445-458.

Notton, D.G. 2008. Insects of Mortimer Forest on the Shropshire/Herefordshire border. Br. J. Ent. Nat. Hist., 21:187-192.

Novak, T. 2005. Terrestrial fauna from cavities in northern and central Slovenia, and a review of systematically ecologically investigated cavities. Acta Carsologica 34(1): 169-210.

Nuvoli, M. T.; Loru, L.; Pantaleoni, R. A. 2007. Elenco preliminare degli insetti dell’isola dell’Asinara (Sardegna NW). Annali del Museo Civico di Storia Naturale “G. Doria”, 98: 425-549.

Ohl, M. 2009. A revision of the mantispid genus Nampista (Neuropterida, Mantispidae). Zoosystematic Evolution 85(2):189-198.

Olsen, K. M. 2008. Noteworthy records of reshwater insects in Norway. Norwegian Journal of Entomology 55:53-71.

Onar, N.; Aktaç, N. 2002. Edirne yöresi Chrysopidae (Neuroptera) faunasi üzerine taksonomik ve faunistik araştirmalar [Tacsonomic [sic] and faunistic studies on the Chrysopidae (Neuroptera) fauna of Edirne province]. Turkiye Entomoloji Dergisi 26(2):121-134.

Pagola Carte, S. 2007. Inventario y seguimiento de la entomofauna del hayedo de Oieleku (Oiartzun, Parque Natural de Aiako Harria). Campaña 2007. Asociacion Gipuzkoana de Entomologia, 100 pp.

Pagola Carte, S. 2008. Inventario y seguimiento de la entomofauna del hayedo de Oieleku (Oiartzun, Parque Natural de Aiako Harria). Campaña 2008. Asociacion Gipuzkoana de Entomologia, 98 pp.

Pantaleoni, R. A.; Aspöck, U.; Cao, O. V.; Aspöck, H. 2004. Subilla principiae n. sp., a new spectacular snakefly from Sardinia (Raphidioptera Raphidiidae). Redia 87: 3-6.

Pantaleoni, R. A.; Badano, D. 2012. Myrmeleon punicanus n. sp., a new pit-building antlion (Neuroptera Myrmeleontidae) from Sicily and Pantelleria. Bulletin of Insectology 65 (1): 139-148.

Pantaleoni, R. A.; Badano, D.; Aspöck, U.; Aspöck, H. 2013. Ascalaphus festivus (Rambur, 1842) in Sardinia, a new genus of Ascalaphidae for Europe (Neuroptera). Biodiversity Journal 4(1): 179-182.

Pantaleoni, R. A.; Cesaroni, C.; Nicoli Aldini, R. 2010. Myrmeleon mariaemathildae n. sp.: a new Mediterranean pit-building antlion (Neuroptera Myrmeleontidae). Bulletin of Insectology 63(1): 91-98.

Pantaleoni, R. A.; Fontana, P. 2005. Libelloides longicornis (Linné, 1764) (Insecta, Neuropterida, Planipennia, Ascalaphidae). Memorie del Museo Civico di Storia Naturale di Verona. 2ª serie. Monografie Naturalistiche 2: 145-148.

Paulian, M. 2002. Recent additions to the green lacewing fauna of Romania (Neuroptera: Chrysopidae). Acta Zoologica Academiae Scientiarum Hungaricae 48 (Suppl. 2), 265-269.

Paulian, M.; Canard, M. 2000. Green lacewings new to the fauna of Rumania (Neuroptera: Chrysopidae). Entomofauna 21(20):249-252.

Paulian M., Canard M., Thierry D., Ciubuc C., 2001. Survey of green lacewings in southern Transylvania, Rumania, with some ecological notes (Neuroptera: Chrysopidae). J. Neuropt. 3:25-31.

Paulian, M.; Viorica, M. 2001. Les arthropodes prédateurs dans les pechers du sud-ouest d'Oltenia, Roumanie [=The predatory arthropds of peach orchards from southeast of Oltenia, Romania]. Analele Institutului de Cercetari Pentru Protectia Plantelor 28:63-69.

Pawłowski, J., 2003. Invertebrates. In: Witkowski, Z. J. et al. (eds), 2003. Carpathian list of endangered species. WWF and Institute of Nature Conservation, Polish Academy of Sciences, Vienna, Krakow, 64 pp.

Pérez, A.J.; Morales, E.; Oromí, P.; López, H. 2003. Fauna de artrópodos de Montaña Clara (islas Canarias) II: Hexápodos (no coleópteros). Vieraea 31: 237-251.

Perovic, F.; Britvec, B.; Milosevic, B.; Harapin, M. 2005. Die Insecktensammlung von Andrija Hensch. Linzer biol. Beitr., 37(1): 107-112.

Petrovic, S. 2013. A contribution to the knowledge of the Neuroptera (Insecta) fauna of Serbia. Biologica Nyssana 4(1-2):93-96.

Plant, C. 2001. Snake-flies, alderflies and lacewings (Neuropterida) and scorpion-flies (Mecoptera): a provisional review of their status and distribution in Essex with notes on their etymology. Essex Naturalist (N.S.) 18:177-218.

Plant, C. W.; Harvey, M.C. 2008. Neuroptera. Br. J. Ent. Nat. Hist., 21:185-186.

Polevoi, A.V.; Humala, A.E.; Gorbach, V.V.; Uzenbaev, S.D. 2009. Changes and additions to the list of rare and vulnerable insect species of Republic of Karelia . Trudy Karel'skogo nauchnogo tsentra 1: 90-97.

Ponel, P.; Papazian, M. 2003. Une belle località à Odonates en Sardaigne: le lac Baratz. Martinia 18(3): 93-96.

Popov, A. 2000. Entomofaunistic diversity of the Central Balkan National Park. Orthopteroidea, Neuropteroidea, Mecoptera. In: Sakalian M. (ed.). Biological diversity of the Central Balkan National Park. Sofia, Pensoft, 319-362, 491-512, 540-542, 571-573.

Popov, A. 2000. Entomofaunistic diversity of the Rila National Park. Orthopteroidea, Neuropteroidea, Mecoptera. In: Sakalian M. (ed.). Biological diversity of the Rila National Park. Sofia, Pensoft, 285-331, 429-464, 527-531, 581-584.

Popov, A. 2001. The snakeflies and the lacewing insects (Insecta: Raphidioptera and Neuroptera) of the Kresna Gorge (SW Bulgaria). In: Beron P. (ed.). Biodiversity of Kresna Gorge (SW Bulgaria). Sofia, Nat. Mus. Natur. Hist., Inst. Zool., 131-143.

Popov, A. 2002. Neuropterida of northern Europe. Acta Zoologica Academiae Scientiarum Hungaricae 48 (Suppl. 2), 281-291.

Popov, A. 2002. Rare Chrysopidae and Hemerobiidae (Neuroptera) from Bulgaria. Hist. nat. bulg., Sofia, 14: 73-78.

Popov, A. 2004. The Ascalaphidae (Neuroptera) of the Balkan Peninsula. In: Aspöck, U. (eds.): Entomologie und Parasitologie - Festschrift zum 65. Geburtstag von Horst Aspöck, 640 pp. Denisia 13: 229-237.

Popov, A. 2004. List of Raphidioptera, Neuroptera and Mecoptera published from the Eastern Rhodopes (Bulgaria and Greece). In: Beron P., A. Popov (eds). Biodiversity of Bulgaria. 2. Biodiversity of Eastern Rhodopes (Bulgaria and Greece). Sofia, Pensoft, Nat. Mus. Natur. Hist., 371-378.

Popov, A. 2007. Distribution of the families of Neuroptera with low species diversity in Bulgaria. Ann. Mus. civ. St. nat. Ferrara, 8: 117-130.

Popov, A. Letardi, A. 2010. Comparative zoogeographical analysis of Neuropterida of the Apennine and Balkan peninsulas. In: Devetak, D.; Lipovsek, S.; Arnett, A.E. (eds) 2010. Proceedings of the 10th Int. Symp. on Neuropterology. Maribor, Slovenia: 239-256.

Prisny, A. V. 2004. The Ant Lions (Neuroptera: Myrmeleontidae) of the Belgorod Region [Russia]. The Kharkov Entomological Society Gazette 2004 (2005) 12(1–2): 25-27.

Pröse, H.; Gruppe A. 2003. Rote Liste gefährdeter Netzflügler (Neuropteroidea) Bayerns. Schriftenreihe des Bayerischen Landesamtes für Umweltschutz 166: 95-98.

Rausch, H.; Aspock, H.; Aspock, U. 2004. Calabroraphidia renate n. gen., n. sp. – eine neue spezies und ein neues genus der familie Raphidiidae aus Süditalien (Neuropterida, Raphidioptera). Entomologische Nachrichten und Berichte 48(3/4): 159-165.

Rausch, H.; Weissmair, W. 2007. Sisyra bureschi nov. sp. und S. corona nov. sp. – zwei neue schwammhafte und beiträge zur faunistic der Sisyridae (Insecta, Neuroptera) Südosteuropas. Linzer Biol. Beit., 39(2): 1129-1149.

Reddersen, J.; Secher Jensen, T. 2002. The Syrphidae, Coccinellidae, and Neuroptera (s.lat.) of a large Danish spruce forest. Entomologiske Meddelelser 70:113-127.

Reutz, M.; Aistleitner, U. 2003. Beitrag zur kenntnis der ameisenjungfern in Vorarlberg/Austria occ. (Neuroptera, Myrmeleontidae). Vorarlberger Naturschau 12: 83-89.

Riddiford, N. (ed.) 2005. TAIB project S’ Albufera: a Mediterranean model for the study of biodiversity and environmental change. The Albufera International Biodiversity Group Annual Report, 126 pp.

Röhricht, W. 2000. Netzflügler und Schnabelfliegen aus Mitteldeutschland (1. Nachtrag). Naturw. Beiträge Museum Dessau 12:82-92.

Röhricht, W. 2004. Rote list der netzflügler i. w. S. (Neuropterida) des landes Sachsen-Anhalt. Ber. Landes. Umwelt. Sachsen-Anhalt 39: 249-251.

Rudnik, K. ; Gruppe, A. 2005. Contribution to the Neuropterida of Mecklenburg-Vorpommern (Raphidioptera, Megaloptera, Neuroptera: Chrysopidae, Hemerobiidae, Myrmeleontidae). Galathea 18 suppl.: 23-32.

Rudnik, K. ; Hoppe, H. ; Gruppe, A. 2005. Zur Verbreitung der Neuroptera in Mecklenburg Vorpommern einschließlich der Insel Rügen. Nachrichten der Deutsche Gesellschaft für allgemeine und angewandte Entomologie e.V. 19(3):138.

Saure, C. 2005. Rote Liste und gesamartenliste der kamelhalsfliegen, schlammfliegen und netzflügler (Raphidioptera, Megaloptera, Neuroptera) von Berlin. 15 pp. In: Der landesbeauftragte für naturschutz und landschaftspflege / Senatsverwaltung für stadtentwicklung (eds): Rote Listen der gefährdeten Pflanzen und Tiere von Berlin. CD-Rom.

Schertenleib, A.; Haenni, J.-P. 2002. Un individu de Mantispa aphavexelte Aspöck & Aspöck trouvé en Suisse (Planipennia, Mantispidae). Bulletin Romand d’entomologie 20:29-35.

Shortall, C. R.; Plant, C. W. 2012. Nineta inpunctata (Reuter, 1894)(Neu.: Chrysopidae): a second British Isles record. Entomologist’s Rec. J. Var., 124: 234-236.

Skolka, M. 2008. Invertebrate diversity in the western part of Black Sea coast: Cape Midia-Cape Kaliakra. 90-110. In: Fagaras, M. (coord.) 2008. Studii comparative privind biodiversitatea habitatelor costiere, impactul antropic şi posibilităţile de conservare şi restaurare a habitatelor de importanţă europeană dintre Capul Midia şi Capul Kaliakra - Volum cu lucrările Conferinţei de la Constanţa (Mamaia, 26-28 septembrie 2008), 161 pp.

Sumpich, J.; Kacirek, A. 2006. Contribution to the knowledge of ant-lions (Neuroptera: Myrmeleontidae) of Ceskomoravska vrchovina highlands. Klapalekiana 42: 327-330.

Szentkirályi, F.; Kazinczy, L.; Kadar, F.; Bernath, B.; Barabas, S. 2007. Monitoring of Antlions (Neuroptera Myrmeleontidae) by light trapping: influence of weather elements on daily and seasonal flight patterns. Ann. Mus. civ. St. nat. Ferrara, 8: 167-172.

Sziráki, G. 2002. Neuropteroidea (Megaloptera, Raphidioptera, Neuroptera) from the Fertő-Hanság National Park. In: The fauna of the Fertő-Hanság National Park: 537-543.

Sziráki, G. 2007. Presence of the subgenus Xeroconiopteryx Meinander, 1972 in Hungary (Neuroptera: Coniopterygidae). Annales Historico-Naturales Musei Nationalis Hungarici 99: 93-100.

Sziráki, G. 2008. The presence of Nevrorthidae Nakahara family, 1915 [sic] (Insecta: Neuroptera) in the Cernei Mountains. Acta Siculica 2008: 87-90.

Tannert, R. F. 2002. Erfassung der Insektenfauna im Nürnberger Reichswald bei Fishbach-Brunn von 1978 bis 1999. Galathea 18:67-68.

Tannert, R. F. 2003. Erfassung der Insektenfauna im Nürnberger Reichswald bei Fishbach-Brunn 2002 und 2003. Galathea 19:137-151.

Tannert, R. F. 2007. Erfassung der Insektenfauna im Nürnberger Reichswald bei Fishbach und Brunn 2004 - 2006. 4. Nachtrag. Galathea 23(1):9-24.

Tannert, R. F. 2007. Erfassung der Insektenfauna im Nürnberger Reichswald an der gastrasse zwischen Buchenbühl und Autobahn A3 in der jahren 2004 - 2006. Galathea 23(2):45-74.

Tannert, R. F. 2013. Erfassung der Insekten- und Spinnenfauna westlich von weissenbrunn/Mfr. und ernhofen/Mfr. im bereich der waldabteilung wolfsgrube und angrenzenden lebensräumen mit hochspannungs-trasse, pappelbestand, früheren sandgruben, hochstaudengesellschaften incl. NSG „Flechten-kiefernwälder südl. L:einburg“ - TK 25, Nr.6534 - 1. Nachtrag aus den Jahren 2008 bis 2012. Galathea 29:23-41.

Tannert, R.; Dunk, K. von der 2004. Erfassung der Insektenfauna im jetzigen NSG “Tennenloher Forst” östlich Tennenlohe bei Erlangen/Mittelfranken. Galathea 20:125-147.

Tannert, R.; Rupprecht, R. 2000. Erfassung der Insektenfauna im Nürnberger Reichswald bei Fishbach-Brunn von 1978 bis 1999 – insbesondere Macro-, Microlepidoptera und Coleoptera. Galathea 16:75-108.

Tannert, R.; Dunk, K. von der 2008. Erfassung der Insektenfauna westl. von weissenbrunn und ernhofen/Mfr. im bereich der waldabteilung wolfsgrube und angrenzenden lebensräumen mit hochspannungs-trasse, pappelbestand, früheren sandgruben, hochstauden-gesellschaften incl. NSG „Flechten-Kiefernwälder südlich Leinburg“ in den jahren 2000 bis 2007. Galathea 24(4):157-223.

Thaler, K. 2004. Fragmenta faunistica Tirolensia – XVII (Arachnida: Araneae; Insecta: Psocoptera, Strepsiptera, Megaloptera, Neuroptera, Raphidioptera, Mecoptera, Siphonaptera, Diptera: Mycetophiloidea). Veröffenlichungen des Tiroler Landesmuseums Ferdinandeum 84: 161-180.

Thaler, K. 2005. Fragmenta faunistica Tirolensia – XVIII (Arachnida: Araneae, Pseudoscorpiones; Diplopoda: Julida; Insecta: Rhynchota [Heteroptera, Cicadina, Coccina, Aleyrodina, Psyllina, Aphidina], Planipennia [Myrmeleontidae], Diptera [Psychodidae]). Veröffenlichungen des Tiroler Landesmuseums Ferdinandeum 85: 279-297. (figura)

Thunes, K. H., Skartveit, J., Gjerde, I., Starý, J., Solhøy, T., Fjellberg, A., Kobro, S., Nakahara, S., zur Strassen, R., Vierbergen, G., Szadziewski, R., Hagan, D.V., Grogan Jr., W. L., Jonassen, T., Aakra, K., Anonby, J., Greve, L., Aukema, B., Heller, K., Michelsen, V., Haenni, J.-P., Emeljanov, A. F., Douwes, P., Berggren, K., Franzen, J., Disney, R. H. L., Prescher, S., Johanson, K. A., Mamaev, B., Podenas, S., Andersen, S., Gaimari, S. D., Nartshuk, E., Søli, G. E. E., Papp, L., Midtgaard, F., Andersen, A., von Tschirnhaus, M., Bächli, G., Olsen, K. M., Olsvik, H., Földvári, M., Raastad, J. E., Hansen, L. O., Djursvoll, P. 2004. The arthropod community of Scots pine (Pinus sylvestris L.) canopies in Norway. Entomologica Fennica 15: 65-90.

Tillier, P. 2006. Présence de Nineta pallida (Schneider, 1851) dans les Cévennes (Neuroptera Chrysopidae). L’Entomologiste 62(5/6): 187.

Tillier, P. 2008. Présence de Dichochrysa venusta (Hölzel, 1974) en Haute-Balagne : nouvelle espece pour la Corse et deuxieme donee pour la France (Neuroptera Chrysopidae). L’Entomologiste 64(4): 253-254.

Tillier, P. 2008. Inventaire entomologique du marais de Stors (Meriel - Val-d’Oise): Mecoptera et Neuroptera. Agence des Espaces Verts de la Région Ile-de-France, 6pp.

Tillier, P. 2008. Inventaire entomologique du site du Bois de la Tour du Lay (Val-d’Oise): Mecoptera et Neuroptera. Conseil Général du Val-d’Oise, 5pp.

Tillier, P. 2008. Inventaire entomologique du marais du Rabuais (Val-d’Oise): Mecoptera et Neuroptera. Conseil Général du Val-d’Oise, 4pp.

Tillier, P. 2009. Nouvelle capture de Coniopteryx (Metaconiopteryx) arcuata Kis, 1965 en France et liste actualisée des Coniopterygidae de France (Neuroptera Coniopterygidae). L’Entomologiste 65(5) : 285-286.

Tillier, P. 2010. Capture en Corse de Neuroleon microstenus (McLachlan, 1898) nouvelle espèce pour la France, et nouvelles données sur des fourmilions rares ou peu connus en France (Neuroptera Myrmeleontidae). L’Entomologiste 66(2): 73-80.

Tillier, P. 2010. Hemerobius (Brauerobius) marginatus Stephens, 1836 dans le Val-d’Oise (Ile-de-France) (Neuroptera Hemerobiidae). L’Entomologiste 66(4): 235-236.

Tillier, P. 2011. Micromus (Nesomicromus) lanosus (Zeleny, 1962) dans le Masif central: premières donées francaises en dehors des Alpes et des Pyrénées (Neuroptera Hemerobiidae). L’Entomologiste 67(2): 111-112.

Tillier, P. 2011. Présence de Neuroleon arenarius (Navás, 1904) dans le département de l’Aude (Neuroptera Myrmeleontidae). L’Entomologiste 67(4): 238.

Tillier, P. 2011. Nouvelle contribution à la connaissance d’Osmylus fulvicephalus (Scopoli, 1763) en Île-de-France (Neuroptera Osmylidae). L’Entomologiste 67(4): 239-240.

Tillier, P. 2011. Nouvelle donnée de Dendroleon pantherinus (F., 1787) pour le Vaucluse (Neuroptera Myrmeleontidae). L’Entomologiste 67(5): 303.

Tillier, P. 2012. Présence d’Hemerobius (Hemerobius) schedli (Hölzel, 1970) dans les Alpes françaises (Neuroptera Hemerobiidae). L’Entomologiste 68(4): 253-254.

Tillier, P. 2012. Drepanepteryx algida (Erichson in Middendorff, 1851) dans le Mercantour, espece nouvelle pour la faune de France et liste actualisée des Hemerobiidae de France (Neuroptera). Bulletin de la Societé entomologique de France 117(4): 457-459.

Tillier, P. 2013. Deux espèces du genre Helicoconis Enderlein, 1905, nouvelles pour la France et liste actualisée des Coniopterygidae de France (Neuroptera). Bulletin de la Societé entomologique de France 118(2): 141-144.

Tillier, P. 2013. Nouvelles captures de Raphidia (Raphidia) ligurica Albarda, 1891 et de Phaeostigma (Phaeostigma) italogallica (H. Aspöck & U. Aspöck, 1976) dans le Mercantour (Raphidioptera Raphidiidae). L’Entomologiste 69 (2): 121-123.

Tillier, P. 2013. Présence de Neuroleon arenarius (Navás, 1904) dans le département du Lot-et-Garonne: première donnée pour la France en dehors de la zone méditerranéenne stricte (Neuroptera Myrmeleontidae). L’Entomologiste 69(2): 126.

Tillier, P.; Almeida, J. M.; Cloupeau, R. 2010. Isoscelipteron glaserellum (U. Aspöck, H. Aspöck & Hölzel, 1979), espèce nouvelle pour la faune du Portugal (Neuroptera, Berothidae). Bulletin de la Societé entomologique de France 115(2):193-202.

Tillier, P.; Giacomino, M.; Colombo, R. 2013. Atlas de repartition des fourmilions en France. Revue de l’Association Roussillonnaise d’Entomologie 23 (Suppl.): 1-52.

Tröger, E. J. 2002. Netzflügler (Neuropterida) in Franken. Galathea 13 suppl.: 37-69.

Tröger, E. J. 2002. Neuropterologische Neuigkeiten aus Kreta (Insecta, Neuroptera). Galathea 13 suppl.: 71-73.

Tröger, E. J. 2002. Nachtrag zu „Netzflügler (Neuropterida) in Franken“. Galathea 13 suppl.: 75-76.

Tröger, E. J. 2004. Der Fliegentaghaft Psectra diptera (Burmeister 1839) in Deutschland und seine Geschichte (Neuroptera, Hemerobiidae). In: Aspöck, U. (eds.): Entomologie und Parasitologie - Festschrift zum 65. Geburtstag von Horst Aspöck, 640 pp. Denisia 13: 185-192.

Tröger, E. J. 2005. Über Netzflügler (Neuropterida) in Baden-Württemberg. Galathea 21(2): 93-96.

Tröger, E. J. 2005. Netzflügler (Neuropterida) im westlichen Kreta – eine Übersicht. Galathea 18 suppl.: 33-42.

Tröger, E. J. 2005. Über Netzflügler (Neuropterida) in Baden-Würtemberg. Nachrichten der Deutsche Gesellschaft für allgemeine und angewandte Entomologie e.V. 19(3):140.

Tröger, E. J. 2007. Neue Neuropterenfunde aus dem deutschen Südwesten. Nachrichten der Deutsche Gesellschaft für allgemeine und angewandte Entomologie e.V. 21(3): 132-134.

Tröger, E. J. 2007. Altfränkische Spurensuche. Nachrichten der Deutsche Gesellschaft für allgemeine und angewandte Entomologie e.V. 21(3): 135.

Tröger, E. J. 2007. Psectra diptera, auch in Spanien. Nachrichten der Deutsche Gesellschaft für allgemeine und angewandte Entomologie e.V. 21(3): 142-143.

Tröger, E. J. 2009. Die wiederentdeckung von Sialis nigripes Pictet, 1865 (Neuropterida, Megaloptera) in Baden-Württemberg. Nachrichten der Deutsche Gesellschaft für allgemeine und angewandte Entomologie e.V., 23(2):78-81.

Tröger, E. J. 2009. Netzflügler (Neuropterida) an dinkelberg und hochrhein bei Grenzach-Wyhlen – Erste ergebnisse: Coniopteryx drammonti Rousset, 1864 neu für Deutschland. Nachrichten der Deutsche Gesellschaft für allgemeine und angewandte Entomologie e.V., 23(2):81-82.

Ventura, M. A. 2005. Neuroptera. In: Norges, P. A. V.; Cunha, R.; Gabriel, R.; Martins, A. F.; Silva, L.; Vieira, V. (eds) 2005. A list of the terrestrial fauna (Mollusca and Arthropoda) and flora (Bryophyta, Pteridophyta, Spermatophyta) from the Azores. Direcçao Regional do Ambiente and Universidade dos Açores, Horta, Angra do Heroismo and Ponta Delgada, 317 pp.

Vidlicka, L. 2003. Neuroptera of the region Záhorie (Western Slovakia). Acta Rerum. Nat. Mus. Slov., Bratislava 49:99-104.

Vidlicka, L. 2003. Neuroptera of the Muranska Planina Mts I. – site Paseky. Reussia 1 (suppl.1): 241-244.

Vidlicka, L. 2007. Snakeflies (Raphidioptera) of Ostrov Kopac (Bratislava). :141-144.

Vidlicka, L. 2007. Lacewings (Neuroptera) of Ostrov Kopac (Bratislava). :145-150.

Vidlicka, L.; Holusa, J. 2007. Neuroptera, Raphidioptera and Mecoptera assemblages inhabiting young spruce (Picea abies) forests: dominance structure and seasonal activity patterns. Journal of Forest Science 53: 74-81.

Vidlicka, L.; Majzlan, O. 2002. The lacewings (Neuroptera) in the surroundings of the Natural Monument Sivy kamen in the village of Podhradie. Rosalia (Nitra) 16: 107-111.

Vieira, V.; Borges, P.A.V.; Karsholt, O.; Wunderlich, J. 2003. La fauna de artrópodos de la isla de Corvo (Azores): lista actualizada de las especies incluyendo nuevos registros. Vieraea 31: 145-156.

Volkovich, T. A. 2001. Green lacewings (Neuroptera, Chrysopidae) of the “forest on the Vorskla river” Nature Reserve (Belgorod provincie): fauna and ecology. Entomologicheskoe Obozreni 80:368-382.

Weihrauch, F.; Gruppe, A. 2009. Die Neuropterida des Dürnbucher Forstes: auf der suche nach Myrmeleon bore in Bayern. Nachrichten der Deutsche Gesellschaft für allgemeine und angewandte Entomologie e.V., 23(2):84-85.

Weissmair, W. 2010. Sisyra bureschi und S. dalii (Neuroptera, Sisyridae) neu in Südwest-Deutschland und weitere Beiträge zur Faunistik und Ökologie. Entomologische Nachrichten und Berichte 54(3-4): 207-212.
